# Natural Compounds for Preventing Age-Related Diseases and Cancers

**DOI:** 10.3390/ijms25147530

**Published:** 2024-07-09

**Authors:** Mi-Ran Ki, Sol Youn, Dong Hyun Kim, Seung Pil Pack

**Affiliations:** 1 Department of Biotechnology and Bioinformatics, Korea University, Sejong-Ro 2511, Sejong 30019, Republic of Korea; allheart@korea.ac.kr (M.-R.K.); youseul0419@korea.ac.kr (S.Y.); jklehdgus@korea.ac.kr (D.H.K.); 2 Institute of Industrial Technology, Korea University, Sejong-Ro 2511, Sejong 30019, Republic of Korea

**Keywords:** natural compounds, aging, age-related diseases, cancer, autophagy, circadian rhythms

## Abstract

Aging is a multifaceted process influenced by hereditary factors, lifestyle, and environmental elements. As time progresses, the human body experiences degenerative changes in major functions. The external and internal signs of aging manifest in various ways, including skin dryness, wrinkles, musculoskeletal disorders, cardiovascular diseases, diabetes, neurodegenerative disorders, and cancer. Additionally, cancer, like aging, is a complex disease that arises from the accumulation of various genetic and epigenetic alterations. Circadian clock dysregulation has recently been identified as an important risk factor for aging and cancer development. Natural compounds and herbal medicines have gained significant attention for their potential in preventing age-related diseases and inhibiting cancer progression. These compounds demonstrate antioxidant, anti-inflammatory, anti-proliferative, pro-apoptotic, anti-metastatic, and anti-angiogenic effects as well as circadian clock regulation. This review explores age-related diseases, cancers, and the potential of specific natural compounds in targeting the key features of these conditions.

## 1. Introduction

The incidence of cancer in people over 65 is 11 times higher than in younger people, and the statistical fact that more than 60% of the newly diagnosed cancer patients are over 65 suggests that aging and cancer are closely linked [1,2]. The root cause of cancer is the acquisition of somatic mutations in primary oncogenes or tumor suppressor genes [3]. Somatic mutations accumulate over time, so it makes sense that the risk of developing cancer gradually increases with age. Additionally, there is also growing interest in the role of the aging environment in triggering cancer development [4]. But the relationship between the two is more complex. 

The accumulation of cellular damage promotes both cancer and aging [5]. Cellular damage encompasses a range of issues including DNA damage, genetic mutations, cellular senescence, and chronic inflammation [6,7]. It is important to note that the mechanisms protecting cells from damage also have the potential to prevent cancer and aging [5,7]. On the other hand, while cancer and longevity necessitate constant cell proliferation potential, the mechanisms that limit unlimited proliferation can protect against cancer but may promote aging [5,6,7]. Factors such as telomere attrition [8], stem cells [9], apoptosis [10], changes in the immune system [11], and altered metabolic activity [12] all play pivotal roles in elucidating the differences between aging and cancer development. 

Autophagy, a fundamental cellular process, maintains homeostasis and promotes differentiation, development, and survival by utilizing lysosome-mediated degradation to eliminate molecules and intracellular components such as nucleic acids, proteins, lipids, and organelles [13]. When autophagy is compromised, damaged cellular components accumulate. This accelerates cellular aging and functional decline, leading to various age-related diseases such as Alzheimer’s and cardiovascular diseases. Impaired autophagy also affects stem cell function, leading to tissue dysfunction and reduced regenerative capacity, which are hallmarks of aging and contribute to the overall decline in tissue health. There is an increasing amount of preclinical evidence indicating that manipulating autophagy could potentially serve as an inhibitor of age-related diseases, such as neurodegenerative disorders [13]. 

Conversely, circadian clocks are known to regulate several intracellular signaling pathways, including cell proliferation, DNA damage repair and response, angiogenesis, metabolic and redox homeostasis, inflammation, and immune responses [14]. Circadian clocks also regulate the process of autophagy, resulting in variations in both the brain and the periphery. Chaperone-mediated autophagy, for instance, shows a primary core clock protein, BMAL1-dependent rhythms in the liver, heart, and kidney [15]. Based on evidence that circadian rhythms can be influenced by environmental factors, social behaviors, and pre-existing pathological conditions, it has been demonstrated that perturbations in circadian rhythms can affect several physiological processes associated with cancer and inflammatory diseases [14].

Important public health concerns include the aging of the world’s population and the rise in the prevalence of chronic illnesses like diabetes, heart disease, and cancer [16]. In response to these challenges, healthcare systems are transitioning from a focus on treatment to prevention [17]. This shift is being propelled by growing consumer awareness of the potential health benefits of functional foods, dietary supplements, and medical foods [18,19]. Concurrently, there is an increasing preference for alternative therapies and a rising demand for plant-based products [20]. Interestingly, over 40% of the pharmaceutical formulations are derived from natural products and notable drugs such as morphine, artemisinin, and paclitaxel have their origins in natural products [21]. Traditional medicine, which is based on herbal products, has been utilized for centuries to treat a range of illnesses, from minor ailments to major diseases, and to enhance the immune system [22,23,24]. Phytochemicals (“phyto” means plant in Greek word) are the biologically active natural chemical constituents of plants [25]. In the fight against cancer and to live longer, the role of phytochemicals is becoming a more prominent topic of study [26]. Polyphenols, flavonoids, and carotenoids are among the phytochemicals known to support longevity by exerting antioxidant effects that minimize damage to essential macromolecules like DNA, proteins, and organelles [27]. Additionally, they help regulate cell proliferation and senescence by influencing energy metabolism and nutrient-sensing processes that change with age [28]. Beyond their anti-aging properties, phytochemicals have demonstrated potent anticancer effects [29,30]. They are capable of reducing inflammation; modulating the expression of oncogenic or tumor suppressor proteins; regulating the expression of genes involved in cancer initiation, progression, and metastasis; and modulating the immune response. 

This comprehensive review discusses age-related diseases, cancer, and their interrelationships. It underscores the dual role of natural compounds as beneficial health ingredients and promising therapeutic agents in combating geriatric diseases, metabolic disorders, and cancer. The review also emphasizes the growing significance of circadian rhythms, particularly in lifestyle changes, to prevent chronic diseases. It explores the intriguing connections between the disruptions of the circadian clock and these diseases, as well as the potential role of natural compounds in their regulation. By clarifying these important areas, this review emphasizes the need for natural compounds in the treatment and prevention of diseases.

## 2. Circadian Rhythm and Implication for Chronic Diseases

The healthcare system encourages lifestyle modification to prevent and manage chronic illness as the prevalence of these conditions increases due to the growing elderly population. Common lifestyle modifications encompass maintaining an optimal body weight, engaging in regular physical exercise, adhering to a nutritious diet, restricting smoking and alcohol intake, undergoing routine health check-ups, managing stress, engaging in mental stimulation, and ensuring sufficient sleep [31,32]. Although the effects of these modifications may differ among individuals due to genetic and environmental influences, adopting lifestyle modification that aligns with natural circadian rhythms, such as maintaining regular sleep and eating patterns, can help optimize our health and well-being [33,34].

**Figure 1:**
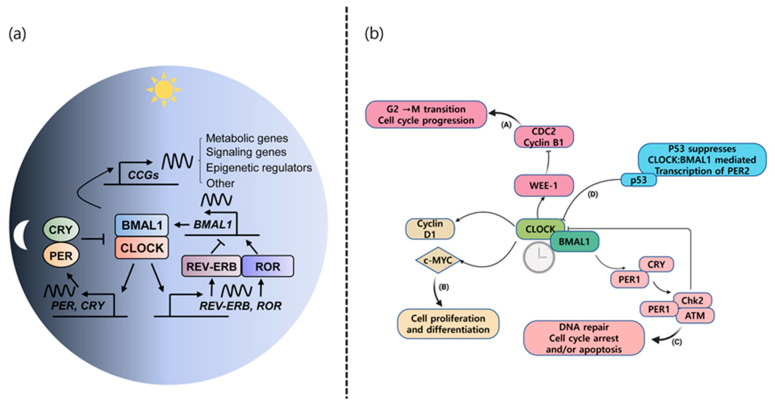
(**a**) This autoregulatory feedback loop cycles between the CLOCK/BMAL1 transcriptional activator complex and its repressors (PER/CRY, REV-ERBα) or activators (RORα/β) to constitute the molecular clock oscillator that drives the expression of multiple clock-controlled genes (CCGs), such as metabolic genes, signaling genes, and epigenetic regulators. Reproduced from Ref. [38] (CC By 4.0). (**b**) Molecular interaction between the circadian core clock and cell cycle components. The CLOCK:BMAL1 complex transcriptionally activates genes containing E-box regulatory elements in their regulatory regions, such as clock genes and cell cycle genes. (A) The CLOCK:BMAL1 complex directly controls the transcription of the cell cycle-related gene Wee-1 which contains three B-boxes in its promoter and encodes a protein kinase that inactivates the CDC2/Cyclin B1 complex, thus regulating G2-M transition and cell cycle progression. (B) Transcriptional activation of the genes encoding Cyclin D1 and C-MYC by CLOCK:BMAL1 affects cell proliferation and differentiation. (C) PER1 can complex with the ATM kinase and the checkpoint kinase Chk2, thus impinging upon DNA repair, cell cycle arrest, and/or apoptosis. (D) Both physiological and stress-induced p53 binds to the p53 response element in the PER2 promoter, which overlaps with the BMAL1/CLOCK-binding site, thereby inhibiting the CLOCK:BMAL1-mediated transcription of PER2. Reproduced from Ref. [14] (CC By 4.0).

The circadian system of the human body regulates various physiological processes and adapts behavior based on the biological clock in response to daily changes in the environment [35]. The suprachiasmatic nucleus (SCN), located in the anterior hypothalamus of the brain, is the central pacemaker of the circadian timing system and regulates most circadian rhythms in the body [36]. It is synchronized to the 24 h light/dark cycle. This biological clock is made up of proteins encoded by thousands of genes that turn on and off in a specific order by a feedback loop system [37,38]. The two primary clock proteins, circadian locomotor output cycle kaput (CLOCK) and brain and muscle ARNT-like protein 1 (BMAL1), bind in the cytosol of SCN cells in the brain. They then move to the nucleus to bind to the regulatory elements in DNA containing E-boxes, activating the expression of the period (PER) and cryptochrome (CRY) genes, initiating the production of the proteins. The PER and CRY proteins work together and return to the nucleus, where they inhibit their own transcription by affecting the CLOCK-BMAL1 pair. This process repeats daily to maintain our body’s circadian rhythm. In an additional feedback loop, the CLOCK-BMAL1 heterodimer also activates the expression of other nuclear receptors, REV-ERB α and RORα [39]. The REV-ERB α protein represses the transcription of BMAL1, while RORα activates it through their competition at specific response elements (ROREs), thereby regulating the production of BMAL1 [40]. The mammalian molecular clock machinery is shown in Figure 1a. These actions ensure that the entire cycle takes about 24 h to complete, corresponding to our body’s circadian rhythm. Light and darkness exert the most profound influence on circadian rhythms, but other factors such as food consumption, stress, physical activity, social environment (including jet lag and shift work), and temperature also affect these rhythms [41]. In addition to their main role in regulating circadian rhythms, core clock components have been discovered to have essential functions in different intracellular pathways and contribute to other physiological processes [14]. Endogenous cellular clocks regulate the rhythmic expression of genes, with approximately 20% of the genome being under circadian control [14]. Examples of these physiological pathways regulated by the circadian rhythm include cell growth, DNA repair and damage, angiogenesis, autophagy, apoptosis, metabolism, redox status, as well as immune and inflammatory processes [42,43,44,45]. Disruptions in these biological rhythms have been correlated with a shorter lifespan in aged mice [46] and heightened susceptibility to cardiovascular disease and metabolic disorders [47], and have been connected to the process of aging, increased risk of cancer, and its development and advancement [48,49]. Multiple epidemiological studies have indicated that engaging in night shift work or experiencing chronic jet lag may increase the risk of developing common forms of cancer, including breast, lung, prostate, colorectal, and skin cancers [50]. The International Agency for Research on Cancer (IARC) classified “shift work that causes disturbances in the body’s natural rhythm” as potentially “causing cancer in humans (Group 2A)” in both 2007 and 2019 based on the available evidence [38,51]. These findings indicate that preserving the integrity of our circadian rhythms may be essential for our overall well-being.

## 3. Aging-Related Disease

As life expectancy increases, so do the prevalence and a significant portion of deaths from age-related diseases such as ischemic heart disease, stroke, other cardiovascular diseases, diabetes, and Alzheimer’s disease (Table 1). The mortality rate for age-related diseases among individuals aged 50 and above is more than twofold to fourfold greater than that for the general population (Table 2). 

### 3.1. Cardiovascular Diseases

Cardiovascular diseases (CVDs), the leading cause of death worldwide, prevail in two-thirds of the people over the age of 60 and more than 85% of the people over the age of 85 [52]. As people age, their hearts and blood vessels undergo changes, resulting in the increased stiffness of the heart and alterations in the filling of blood [53]. These changes can have an impact on heart health, even in the absence of heart disease. The typical indications of an aging heart comprise a reduction in heart size, modifications in the heart’s outer layer, as well as enlarged ventricles and aortas [52]. Next, we look at the four major age-related CVDs: coronary heart disease, hypertension, heart failure, and stroke. Patients with CVD have a higher risk of developing various types of cancer and experiencing cancer-related mortality, although variation exists among studies and among types of cancer [54]. Common mechanisms such as chronic inflammation, oxidative stress, metabolic dysregulation, the clonal hematopoiesis of uncertain potential, microbial dysbiosis, hormonal effects, and circadian disruption and cellular aging underlie both CVD and cancer. Understanding these shared risk factors and mechanisms is crucial for predicting, preventing, and treating both diseases, and is essential for advancing the field of cardio-oncology [54]. Many drugs for cancer treatment and radiation therapies can be harmful to the heart, leading to conditions such as heart failure, arrhythmias, coronary artery disease, hypertension, and other CVDs [55]. 

**Table 1:** Top 10 causes of death and deaths per 100,000 population in 2019.

Rank	Global	USA	Republic of Korea
1	Ischemia heart disease (115.7)	Ischemia heart disease (153.4)	Ischemia heart disease (54.8)
2	Stroke (80.7)	Alzheimer’s disease and other dementias (87.3)	Lower respiratory infection (52.0)
3	Chronic obstructive pulmonary disease (42.0)	Chronic obstructive pulmonary disease (59.4)	Stroke (50.0)
4	Lower respiratory infection (33.8)	Stroke (48.2)	Trachea, bronchus, and lung cancers (39.6)
5	Neonatal condition (26.5)	Trachea, bronchus, and lung cancers (47.3)	Self-harm (28.6)
6	Trachea, bronchus, and lung cancers (23.2)	Kidney diseases (26.1)	Alzheimer’s disease and other dementias (23.7)
7	Alzheimer’s disease and other dementias (21.4)	Drug use disorders (22.6)	Liver cancer (22.6)
8	Diarrheal diseases (19.8)	Hypertensive heart disease (20.0)	Colon and rectum cancers (19.9)
9	Diabetes mellitus (19.5)	Colon and rectum cancers (18.8)	Kidney diseases (19.7)
10	Kidney diseases (17.4)	Diabetes mellitus (18.7)	Stomach cancer (16.9)

Global Health Estimates 2020: Deaths by Cause, Age, Sex, by Country and by Region, 2000–2019. Geneva, World Health Organization; 2020. The number in parentheses represents the number of deaths per 100,000 people. Adapted from [56,57]. © Copyright World Health Organization (WHO), 2021.

**Table 2:** Top 10 causes of death and deaths per 100,000 population among 50+ age in 2019.

Rank	Global	USA	Republic of Korea
1	Ischemia heart disease (455)	Ischemia heart disease (422)	Ischemia heart disease (136)
2	Stroke (320)	Alzheimer’s disease and other dementias (247)	Lower respiratory infection (133)
3	Chronic obstructive pulmonary disease (174)	Chronic obstructive pulmonary disease (167)	Stroke (124)
4	Trachea, bronchus, and lung cancers (93)	Stroke (132)	Trachea, bronchus, and lung cancers (100)
5	Lower respiratory infection (90)	Trachea, bronchus, and lung cancers (131)	Alzheimer’s disease and other dementias (61)
6	Alzheimer’s disease and other dementias (90)	Kidney diseases (71)	Liver cancer (55)
7	Diabetes mellitus (76)	Hypertensive heart disease (53)	Kidney diseases (50)
8	Kidney diseases (63)	Colon and rectum cancers (50)	Colon and rectum cancers (49)
9	Hypertensive heart disease (60)	Lower respiratory infection (49)	Chronic obstructive pulmonary disease (43)
10	Cirrhosis of the liver (53)	Diabetes mellitus (49)	Self-harm (42)

Global Health Estimates 2020: Deaths by Cause, Age, Sex, by Country and by Region, 2000–2019. Geneva, World Health Organization; 2020. The number in parentheses represents the number of deaths per 100,000 people. Adapted from [56,57]. © Copyright World Health Organization (WHO), 2021.

#### 3.1.1. Coronary Heart Disease (CHD)

Coronary Heart Disease, also known as coronary artery disease, is caused by the narrowing of the coronary arteries due to plaque buildup. This buildup, often a result of atherosclerosis, leads to reduced blood flow to the heart muscle [58]. As a result, the heart may not receive the oxygen and nutrients it needs to function effectively, leading to symptoms such as chest pain and shortness of breath. CHD is also called ischemic heart disease because it is a condition where narrowed blood vessels do not get enough blood and oxygen to the heart. CHD is a major contributor to various other heart conditions and can lead to serious complications, including heart attacks. Seriously, this is the world’s biggest killer in 2019 around the globe by the report of WHO (Table 1 and Table 2). High blood pressure, unhealthy blood fats, sugar problems, and being overweight are key health issues that can lead to heart disease in older people, with diabetes becoming a bigger concern, especially for women [59,60].

#### 3.1.2. Hypertension

As we age, hypertension, or high blood pressure, is a major risk factor for CVDs that are the leading cause of death from hypertension [61]. It is a condition where the force of the blood against the artery walls is too high. Persistent hypertension can lead to other cardiovascular complications, including heart disease and stroke. It was estimated that in 2015, 14% of all the deaths were attributed to a systolic blood pressure exceeding 140 mm Hg [62,63]. Yet, individuals with a systolic blood pressure of 110 to 115 mm Hg or higher are generally considered to have a minimal risk level of blood pressure [63]. Risk factors for hypertension include older age, being overweight or obese, the lack of physical activity, a high-salt diet, low potassium intake, and excessive alcohol consumption [64]. High blood pressure is a major risk factor for developing atherosclerosis, the main cause of CVD [65]. High blood pressure is linked to tumor-like mechanisms as it affects artery walls through oxidative stress and is associated with the development of cellular cancer [66]. Research indicates that individuals with high blood pressure have an increased risk of mortality from cancer, particularly renal cell carcinoma [67].

#### 3.1.3. Heart Failure

Heart failure (HF) occurs when the heart is unable to pump enough blood to meet the body’s needs, leading to symptoms such as fatigue, shortness of breath, and fluid retention [68]. The aging population is a key factor driving this trend, with HF frequently being secondary to coronary artery disease [68]. Additionally, given the associations of HF with conditions such as hypertension, type 2 diabetes, chronic inflammation, coronary artery disease, sarcopenia, and obesity, it is crucial to explore nutritional recommendations for the prevention and treatment of HF in order to enhance clinical outcomes [69]. Experimental studies have demonstrated that heart failure may increase the risk of developing tumors by releasing specific heart failure-related proteins, like SERPINA3, into the bloodstream. These proteins can contribute to the development and growth of tumors. Additionally, elevated levels of cardiac and inflammatory markers have been associated with the onset of new cancers [70].

#### 3.1.4. Stroke

A stroke occurs when blood flow to the brain is disrupted, leading to damage to the brain cells. This disruption can result from a blockage by blood clots in a blood vessel (ischemic stroke) or from a ruptured blood vessel by high blood pressure and the overuse of anticoagulants (hemorrhagic stroke) [71]. This can cut off the supply of oxygen and nutrients to brain tissue, causing damage to brain cells. As a result, stroke can cause severe speech and behavioral problems and even death. Hypertension, diabetes mellitus, alcohol consumption, and obesity have been identified as the factors significantly contributing to the modifiable risk of stroke [72,73]. In addition, the non-modifiable risk factors include a family history of stroke, age, sex, and race/ethnicity [74]. Stroke prevention includes efforts such as quitting smoking, eating healthy, staying active, and controlling weight, but is specifically focused on preventing strokes before they happen and managing conditions such as high blood pressure and diabetes [74]. Cancer can increase stroke risk through direct effects, blood clotting issues, or infections. Treatments like chemotherapy also raise stroke chances [66]. 

The circadian rhythm of blood pressure causes natural fluctuations over a 24 h period, resulting in higher blood pressure during the day and lower blood pressure at night. In individuals with normal circadian rhythms, blood pressure tends to decrease by 10–20% during nighttime sleep, known as “dipping”. However, persistent circadian disruption from irregular work schedules or frequent time zone changes can weaken these fluctuations [75]. Ramsey et al. explored the effects of chronic circadian disruption on the development of stroke in hypertensive rats, finding that a phase progression schedule accelerated stroke and timed feeding delayed stroke [75]. Blood pressure circadian rhythms were weakened by persistent circadian disruption, increasing the risk of cardiovascular complications in the presence of cardiovascular risk factors. The dysregulation of circadian rhythms can lead to the activation of inflammatory processes at specific times of the day, thereby increasing the risk of thrombosis and the progression of atherosclerosis [76]. Atherosclerosis, which is a chronic inflammatory disease, is initiated by endothelial dysfunction and the upregulation of adhesion molecules [77]. Emerging evidence indicates that disrupted circadian rhythms may accelerate the progression of atherosclerosis by regulating endothelial function [78]. In unstable plaques, CLOCK expression and autophagy were found to be decreased compared to stable plaques in both dataset analysis and human carotid atherosclerotic plaque samples. This suggests that the downregulation of CLOCK may impair endothelial cell autophagy and accelerate atherosclerotic plaque. In the active-phase male mouse stroke model, circadian rhythms influence stroke outcomes by regulating GluA1 expression and autophagic activity [79]. In the active phase model, it was observed that the inhibition of autophagy led to an increase in infarct volume, while its induction resulted in a decrease. Furthermore, the expression of GluA1, a protein involved in synaptic plasticity, was found to decrease with the activation of autophagy and increase when autophagy was inhibited. This indicates a potential inverse relationship between autophagy activity and GluA1 expression. Interestingly, *Per1* gene elimination resulted in the disappearance of the circadian rhythm of infarct volume, GluA1 expression, and autophagy activity, emphasizing its significance in modulating the pathophysiological mechanisms of stroke. The clinical manifestations of CVD also exhibit circadian variation, with a higher incidence in the early morning [80]. This pattern aligns with circadian oscillations in circulating parameters such as hormones and white blood cell counts [81]. Understanding the impact of circadian rhythms on these parameters may provide insights into why myocardial infarction often occurs in the early morning [80]. The depletion of REV-ERBs in cardiomyocytes leads to the development of age-onset dilated cardiomyopathy, a condition characterized by enlarged and weakened heart muscles, ultimately resulting in premature death [82]. This suggests that targeting the circadian REV-ERBs, specifically focusing on their regulatory role in repressing E4bp4, could be a promising therapeutic strategy [82]. 

Finally, age-related CVDs pose a significant health challenge. Understanding these conditions and their risk factors is crucial for prevention and management. Regular check-ups, maintaining a healthy lifestyle, and managing existing health conditions are key to reducing the risk of these diseases [83,84]. 

### 3.2. Degenerative Brain Disorders

Brain aging is a complex process that affects everything from the cellular to the organ level, beginning early in life and accelerating with age [85]. Morphologically, brain aging is mainly characterized by brain volume loss, cortical thinning, white matter degradation, the loss of gyrification, and ventricular enlargement [86]. Pathophysiologically, brain aging is associated with neuronal shrinkage, dendritic degeneration, demyelination, small vessel disease, metabolic slowdown, microglial activation, and white matter lesion formation. These age-related brain diseases typically manifest as late-onset Alzheimer’s disease in more than 95% of the cases, and the other diseases include Parkinson’s disease [87]. 

Autophagy is a cellular process that involves the breakdown and recycling of its own components. This is vital for maintaining cellular health, particularly in neurons. It aids in the removal of damaged organelles and protein aggregates [88]. However, when autophagy is disrupted, it can lead to neurodegenerative diseases such as Alzheimer’s, Parkinson’s, and Amyotrophic Lateral Sclerosis (ALS). These diseases are associated with defects in the autophagy pathway, resulting in the build-up of toxic proteins and damaged cellular components. As individuals age, the efficiency of autophagy decreases. This decline contributes to the increased susceptibility of neurons to damage and the higher prevalence of neurodegenerative diseases in the elderly. Therefore, modulating autophagy presents a promising therapeutic strategy for neurodegenerative diseases. Enhancing this process could help eliminate harmful substances from neurons and potentially decelerate the progression of these diseases. 

#### 3.2.1. Alzheimer’s Disease

Alzheimer’s disease (AD) is a class of chronic neurodegenerative diseases characterized by mitochondrial dysfunction and increased oxidative stress. People diagnosed with this neurodegenerative disease experience a decline in memory, cognition, and, if it persists, the inability to perform the activities of daily living and the need for assistance from others [89]. The leading cause of AD is aging. As the elderly population grows, the number of people with AD is increasing every year [90]. Over the past decade, the incidence of AD has increased by more than 30% in Korea [91]. In the United States, AD is the second-leading cause of death, surpassed only by ischemic heart disease (Table 1). Globally, AD is one of the leading causes of death as the elderly population continues to grow (Table 2). However, although AD has been studied for a long time, its exact cause and mechanism of development are still unknown. The best way to treat AD is to minimize neuroatrophy and synapse loss to improve memory impairment [90]. Various studies have shown that maintaining brain energy metabolism and maintaining homeostasis so that neurons can function is effective against AD [90,92,93]. The pathology of AD is characterized by the abnormal deposits of the β-amyloid peptides or neurofibrillary tangles of phosphorylated tau protein in the cytoplasm, leading to the atrophy or death of nerve cells [89]. 

Circadian disruption in AD can lead to the dysregulation of the sleep/wake cycle, core body temperature rhythms, and melatonin production, impacting overall brain health. Alterations in the clock-regulation of various physiological processes, such as activity rhythms and temperature fluctuations, are the comorbid features of AD. Disrupted circadian timing can accelerate AD pathogenesis by promoting amyloid deposition, oxidative stress, and cell death, forming a self-reinforcing feedback loop [94].

#### 3.2.2. Parkinson Disease

Another example of a brain-related disease is Parkinson’s disease (PD). Similar to AD, PD is a neurodegenerative disorder that occurs primarily in older adults [95]. The patients diagnosed with PD develop pathological changes, including a persistent reduction in dopaminergic neurons in the substantia nigra pars compacta, decreased dopamine levels in the striatum, and the formation of α-synuclein (αSyn) aggregates in the brain [96]. The persistent reduction in dopaminergic neurons in the brain gradually inhibits thalamic activity and reduces the excitatory capacity received by the motor cortex, which can cause slow movements and limb stiffness in PD patients [97]. The exact mechanism of PD is unknown, but when PD progresses, dopaminergic neurons undergo apoptosis. At this time, the abnormal aggregation of αSyn to form aggregates, increased oxidative stress, mitochondrial dysfunction, and increased neuroinflammation have been linked to PD [98]. Therefore, the drugs targeting PD are mainly aimed at alleviating the active symptoms and having neuroprotective effects [99]. 

Several neurodegenerative disorders including PD have been linked to lysosomal malfunction [100]. The decreased lysosomal proteolytic activity of cathepsins D and cathepsin B results in inefficient αSyn degradation, accelerating its accumulation in neurons, which has been linked to PD development [100]. Certain drugs like rapamycin and lithium can enhance autophagy by inhibiting specific pathways, which helps in clearing harmful protein aggregates in neurodegenerative diseases like Huntington’s and Parkinson’s [88]

The disruption of circadian rhythms has a significant impact on degenerative brain disorders. Research indicates a bidirectional relationship between circadian disruptions and neurodegenerative diseases, where disturbances in circadian rhythms and sleep cycles can exacerbate neurodegeneration, while neurodegenerative diseases can, in turn, disrupt circadian rhythms [101]. Not getting enough sleep at night can lead to accelerated nerve cell damage because waste is not properly removed from the brain. During sleep, the brain’s waste removal system is activated, playing a crucial role in removing waste and toxins from the central nervous system (CNS). The meningeal lymphatic vessels are a key part of this system [102]. The circadian rhythm affects the activity of various oxidative stress enzymes and the concentration of glutathione, peaking at 2 a.m. This increased activity during the night suggests that processes intensify during sleep to maintain the redox balance and limit oxidative stress [103]. Circadian rhythm changes can trigger inflammatory responses and oxidative stress by disturbing the redox balance. Neuroinflammation and oxidative stress accelerate neuronal damage and promote the progression of neurodegenerative diseases [104]. Circadian rhythms are essential for the activation and synchronization of neural networks. When neural networks become dysfunctional due to circadian rhythm changes, cognitive, motor, and sensory functions can be impaired [105,106].

### 3.3. Diabetes Mellitus

Diabetes mellitus, a metabolic disorder characterized by impaired glucose homeostasis and hyperglycemia, is caused by a lack of insulin responsiveness or production [107]. It is classified into four categories: type 1 diabetes (T1DM), type 2 diabetes (T2DM), other specific types, and gestational diabetes. These categories are marked by metabolic disorders resulting from hyperglycemia and insulin deficiency, often presenting precursor symptoms such as polydipsia, polyuria, and weight loss [108]. 

Diabetes not only leads to its own development but also a multitude of complications, affecting multiple organs and leading to various structural and vascular disorders [109]. A total of 45 diseases have been identified that affect multiple organs, with a particular predilection for the eyes, kidneys, heart, and nerves [110]. Adults with diabetes have a 50% elevated mortality risk compared to those without diabetes [111]. The disease’s incidence is growing fastest worldwide, with an estimated 693 million adults affected by 2045 [112]. 

The number of elderly patients is increasing at the fastest rate, with one in three adults over 65 in the USA having diabetes [113]. Aging accelerates changes and reduces insulin sensitivity due to the insufficient compensation of beta cell function during the process of increasing insulin resistance. As a result, the level of insulin secretion decreases by 0.7% with age, further increasing the incidence of diabetes in the elderly [114].

Diabetes is closely related to the incidence of cancer, increasing the risk of malignant tumors. Hyperinsulinemia [115], a result of diabetes progression, has been linked to the development of tumors, increasing the risk of breast cancer, colon cancer, and endometrial cancer [116]. The overall risk of developing cancer in diabetic patients is 1.22 times higher than in individuals without diabetes [117]. 

Common complications include diabetic wounds, chronic non-healing wounds that occur due to diabetes and poor self-healing ability [118]. Diabetic foot ulcers (DFUs), infections of the soft tissue or bone, especially under the malleolus, are a typical example of a diabetic wound. DFU causes ulcers to develop in the foot tissue, and in severe cases, the foot may be amputated [119].

Diabetes is also closely related to the pancreas, which secretes insulin to regulate blood sugar levels [120]. When diabetes develops, the function of the pancreas collapses, leading to the development of cancer [121]. Pancreatic cancer, a high-risk cancer with a poor prognosis, is difficult to detect early due to its lack of significant symptoms. Patients with TD2M and pancreatic cancer exhibit an elevated mortality rate of 53% [122].

Diabetes treatment includes weight loss, lifestyle modifications, dietary control, anti-diabetic drugs, and optional obesity surgery [123]. However, treatment effectiveness varies, and the disease’s improvement largely depends on individual willpower. Anti-diabetic drugs can present diverse side effects, and bariatric surgery can lead to several postoperative complications, necessitating the identification of more effective surgical approaches [124,125].

Circadian rhythms synchronize various physiological functions, and their disruption can impair glucose metabolism and insulin secretion, contributing to T2DM pathogenesis through pancreatic dysfunction, fat deposition, and insulin resistance [126]. Circadian rhythm disruption in T2DM is associated with altered mitochondrial metabolism in skeletal muscle, which could potentially contribute to the development of the disease [127]. Chronic disruption of the circadian clock, combined with a high-fat diet, has been found to contribute to weight gain, increased body fat, and impaired glucose tolerance in both mice and humans [128]. However, reducing dietary fat intake could potentially help mitigate the metabolic effects of circadian disruption without necessitating significant sleep loss [128]. Certain single nucleotide polymorphisms (SNPs) in humans can increase or decrease the metabolic risk of T2DM [129]. SNPs in the circadian gene *Per3* increase lipid metabolism T2DM risk. SNPs in the circadian genes *Clock* and *Cry1*, which regulate lipid metabolism, can also increase T2DM risk. SNPs in the neural PAS domain protein 2 structure, a CLOCK-like protein that binds to BMAL1, are also linked to metabolic syndrome risk factors like high blood pressure. Hypertension, T2DM, hyperglycemia, and gestational diabetes are linked to *Bmal1* SNPs. *Cry2* and *Per2* SNP carriers have impaired glucose tolerance, while *Per2* SNP carriers who overeat and eat stress-relatedly gain weight. Two SNPs in the melatonin receptor 1B (Mtnr1b) gene may also increase T2DM risk. In conclusion, molecular circadian clock SNPs and their genes may significantly affect metabolic syndrome, obesity, and T2DM risk [129].

### 3.4. Musculoskeletal Disorders

The most common musculoskeletal disorders associated with aging include osteoporosis, which leads to a decrease in bone density and an increased risk of fractures; degenerative disc disease, causing the progressive deterioration of the intervertebral discs in the spine, leading to back pain and nerve compression; articular cartilage loss, resulting from cartilage wear or inflammation in the joints; degenerative joint disease and arthritis; and muscle atrophy and sarcopenia, a progressive loss of muscle mass and strength due to a decrease in the number and size of muscle fibers and tissues [130,131]. These conditions can be mitigated with appropriate exercise and lifestyle changes, although aggressive intervention may be necessary for treatment [132,133,134]. Age-related musculoskeletal diseases are primarily associated with degenerative changes and have different mechanisms than malignant diseases such as cancer. Nevertheless, research into the relationship between aging and cancer development continues to be an important topic, as there are multiple pathways through which cellular changes that occur during the aging process can influence cancer development. This link is supported by the report that new musculoskeletal problems in the back or hip are associated with an increased risk of subsequent cancer incidence and death in older adults [135].

#### 3.4.1. Arthritis

Osteoarthritis (OA), a major cause of physical disability in the elderly, affects 73% of the individuals over 55, with women making up 60% of this demographic [136]. OA can impact any joint, but most commonly affects the knees, hips, spine, and small joints of the hands. Its development can be influenced by factors such as joint injury or overuse, advanced age, and obesity.

Different stages of OA present varying clinical manifestations, and if untreated, can lead to muscle atrophy and joint stiffness [137]. The main pathological mechanisms of OA include the degradation of the extracellular matrix, apoptosis, autophagy, and inflammation [138]. The progression of OA is primarily due to aging chondrocytes, mitochondrial dysfunction, epigenetic modifications, and decreased growth factor response [139].

Rheumatoid arthritis (RA), an autoimmune disease, involves the immune system attacking the synovial joint [100]. Both OA and RA prevalence increase with age and share many risk factors such as aging, obesity, smoking, gender, and levels of C-reactive protein [140]. Studies have shown that people with RA have a higher incidence of lung cancer and cardiovascular disease, both associated with advanced age and smoking [141,142].

Recent research has explored the role of circadian rhythms in these conditions. Exposure to the inflammatory cytokine interleukin-1 (IL-1) disrupted circadian rhythms in engineered cartilage, leading to cartilage matrix degradation [143]. Genetically engineered cartilage from mouse-induced pluripotent stem cells (miPSCs) demonstrated protective effects against IL-1-induced cartilage degradation and circadian rhythm disruption. Chronic circadian rhythm disturbance was found to accelerate knee cartilage degeneration in rats, activating the wingless-int (WNT)/β-catenin signaling pathway and leading to OA-like changes in cartilage [144]. The loss of the clock core gene *Bmal1* in fibroblast-like synoviocytes, crucial for maintaining joint health, resulted in increased foot edema, the local infiltration of mononuclear cells, and heightened cytokine production in an arthritis model, highlighting the significant role of the circadian clock in regulating inflammatory arthritis [145]. 

#### 3.4.2. Osteoporosis and Fracture

Osteoporosis (OP), an age-related chronic disease affecting approximately 6.3% of the men and 21.2% of the women over 50 worldwide, is characterized by a decrease in bone mineral density due to the resorption of bone by osteoclasts exceeding its formation by osteoblasts [146]. Risk factors include family history, low body weight, smoking, excessive alcohol consumption, a sedentary lifestyle, certain medical conditions, and the long-term use of specific medications (such as glucocorticoids) [147,148,149]. 

Postmenopausal women are at a higher risk due to decreased estrogen levels leading to accelerated bone loss [150]. Both nutrients and environmental factors have the potential to influence bone mass. The adequate intake of calcium and vitamin D, along with hormone therapy, is crucial for bone health and serves as the primary prevention and treatment option for OP [10]. Additionally, maintaining a healthy weight and engaging in regular exercise can also help prevent OP. While estrogen hormone therapy can prevent rapid bone loss [151], it may increase susceptibility to estrogen-dependent cancers, necessitating cautious use [152]. 

The receptor activator of NF-κB (RANK) ligand (RANKL) plays a crucial role in directing the transformation of monocyte/macrophage lineage cells into osteoclasts, specialized cells responsible for breaking down bone tissue by activating RANKL/RANK signaling pathway. Denosumab, a monoclonal antibody that blocks the activity of RANKL, has demonstrated efficacy in clinical trials and has received approval from the FDA, confirming the viability of this pathway as a possible target for medication development [49].

The two main bone anabolic pathways are parathyroid hormone (PTH) signaling and canonical WNT/β-catenin signaling. Extensive studies have indicated that PTH stimulates bone anabolism through the direct regulation of osteoblast-lineage cells at multiple levels; these include the enhancement of osteoblast activity, stimulation of osteoblast differentiation, attenuation of osteoblast apoptosis, and activation of quiescent bone-lining cells [153]. The WNT/β-catenin signaling system is essential for controlling bone metabolism, particularly in the process of bone growth. This pathway has yielded vital knowledge regarding the role of osteocytes, which are the most abundant cells in bone, in coordinating bone remodeling [154].

The decline in skeletal stem cells (SSCs) with age impairs tissue regeneration, contributing to OP and reduced fracture healing [155,156]. SSCs tend to generate fewer bone and cartilage lineage cells, favoring the production of stromal cell types expressing inflammatory molecules like CSF1. Research suggests that targeting aging SSCs could be crucial for musculoskeletal therapies [156].

Bone tissue exhibits a natural biological rhythm, with formation during the day and resorption at night [157]. The disruption of this rhythm, as seen in shift workers or those with sleep disturbances, can lead to OP. Melatonin secretion, crucial for regulating circadian rhythms, could potentially be utilized in OP treatment by promoting osteoblast proliferation and differentiation while inhibiting osteoclast differentiation [158]. The key rhythmic component, BMAL1, which stabilizes the circadian timekeeping system in mammals [159], has been observed to be reduced in both human and mouse specimens of senile OP [160]. This suggests that the disruption of the circadian clock may play a significant role in age-related bone loss.

Lastly, bone is a common site of metastasis in advanced cancer, with metastases, myxofibrosarcoma, myeloma, and chondrosarcoma being the most common malignant bone lesions in elderly patients [1,161]. Bone metastases contribute significantly to morbidity, often leading to severe pain, limited mobility, pathological fractures, spinal cord compression, bone marrow aplasia, and hypercalcemia [162].

#### 3.4.3. Muscle Wasting

Skeletal muscle wasting, a condition where protein degradation surpasses synthesis, is often triggered by disease, aging, and physical inactivity. The forkhead box O (FOXO) signaling pathway plays a significant role in this process [163]. Muscle atrophy and sarcopenia in the elderly result from a complex interplay of biological mechanisms, including changes in proteostasis, mitochondrial function, extracellular matrix remodeling, and neuromuscular junction function [164]. The dysregulation of non-coding RNAs, such as microRNAs and long non-coding RNAs, is critical in muscle atrophy induced by conditions like heart failure, cancer cachexia, and aging [165].

Age-related sarcopenia is associated with adipose inflammation, lipid accumulation in muscles, mitochondrial dysfunction, and inflammation, leading to a cycle of metabolic dysfunction [166]. Sarcopenic obesity, the concurrent reduction in muscle mass and function with increased adipose tissue, has serious health implications, including increased mortality, comorbidities, and geriatric syndromes [167]. Sarcopenia plays an important role in the development of frailty and functional disability and is associated with increased cardiovascular disease, atherosclerosis, and mortality [168]. Cachexia, a syndrome involving weight loss and muscle wasting due to serious illness or chronic disease, accounts for 20% of all cancer-related deaths [169]. 

The regulation of the 24 h feeding schedule, physical activity, and light-induced sleep/wake cycle affect the peripheral clocks of the musculoskeletal system [35], independent of the SCN clock. Dysregulated circadian rhythms can lead to musculoskeletal atrophy, with disrupted rhythms increasing inflammatory cytokines contributing to muscle wasting [170]. Glucose/insulin metabolism and REV-ERBβ, a key regulator of circadian rhythms and a factor in inflammation, are important factors that may connect circadian disruption to muscle wasting [171]. Exercise, acting as a zeitgeber (time cue), helps reset the circadian rhythm and combat sarcopenia [172].

Research on the Duchenne muscular dystrophy (DMD)^mdx^ mouse model of muscular dystrophy has highlighted the role of the myogenic clock gene *Bmal1* in protecting against myogenic damage, suggesting that enhancing BMAL1 function could potentially alleviate muscular dystrophy and degenerative muscle diseases. This underscores the multifactorial nature of muscle atrophy and sarcopenia, and the significant role of circadian rhythms in these conditions [173]. 

### 3.5. Ophthalmology Disorders

As we age, it is natural for our eyesight to weaken and for us to become more susceptible to certain eye conditions. Age-related issues such as macular degeneration, cataracts, diabetic retinopathy, and glaucoma often lead to vision problems [174]. Age-related macular degeneration is a condition that affects a person’s central vision, and risk factors include being over 50, smoking, having high blood pressure, and eating a diet high in saturated fat [175]. A cataract is a clouding of the lens of the eye caused by the presence of high molecular weight protein aggregates or the disruption of the lens microarchitecture that results in decreased vision [176]. Glaucoma is a disease that damages your eye’s optic nerve. It usually happens when fluid builds up and increases pressure inside the eye [177]. People with diabetes are at a higher risk of blindness compared to those without diabetes [178]. Additionally, older adults with diabetes are significantly more prone to developing glaucoma and cataracts than older adults without diabetes [179]. 

The disruption of the circadian clock, induced by factors like aberrant light exposure, can significantly impact ophthalmological health. Studies have shown that circadian rhythm disruption leads to visual dysfunction, retinal thinning, and photoreceptor degeneration [180]. Light is the most powerful signal that our body relies on to synchronize its internal circadian clock with the external environment. The circadian system, governed by the daily light/dark cycle, plays a crucial role in regulating various physiological processes, including the secretion of melatonin, a hormone essential for maintaining circadian rhythms [181]. The disruption of the circadian clock due to ocular disease can lead to sleep disturbances. Pharmaceutical therapies like melatonin and tasimelteon may help improve sleep quality in patients with visual impairment [182]. Obstructive sleep apnea (OSA) is a chronic condition where the upper airway collapses, causing repeated pauses in breathing, intermittent hypoxia, and awakenings at night [183]. OSA is associated with the development of diabetic retinopathy, retinal vein occlusion, and central serous chorioretinopathy [184]. The disruption of the circadian clock can lead to diabetic retinopathy due to the excessive CLOCK-dependent expression of DEC2, which is a transcription factor regulating the circadian clock in mammals, and vascular endothelial growth factor (VEGF), affecting neovascularization and potentially contributing to ophthalmologic complications [184]. In addition, the diurnal expression of autophagy proteins was found to be disrupted in the retina of diabetic rodents [185]. Both mice and rats exhibited distinct diurnal rhythmicity in autophagy protein levels, but diabetic animals showed a significant reduction and phase shift. These findings suggest potential new treatment strategies for diabetic retinopathy.

### 3.6. Skin Aging

The skin, accounting for over 15% of our body weight, is the largest organ that primarily shields us from external threats, including pathogens, ultraviolet radiation, temperature fluctuations, dehydration, and chemicals [186,187]. As skin ages, the signs of aging appear due to various intrinsic and environmental factors [188]. 

Intrinsic factors include genetics, physical abilities, hormones, and metabolism, while environmental factors encompass photoaging, air pollution, tobacco, and diet [189]. Among these, ultraviolet (UV) radiation, categorized into UVA, UVB, and UVC, poses the most significant risk. UVA, penetrating the lower dermis, is primarily responsible for photoaging, while UVB affects sunburn and skin tumors. UVC, largely absorbed by the ozone layer, has minimal impact on skin aging [190]. 

UV radiation not only induces wrinkles, reduced skin elasticity, dark spots, and pigmentation but also accelerates collagen and elastin breakdown, causes DNA damage, and forms matrix metalloproteases (MMPs) in the skin, altering the extracellular matrix (ECM) [191]. Aging, accelerated by factors like UV, leads to common symptoms such as wrinkles, dry skin, and pigmentation. Wrinkles occur due to decreased skin elasticity and collagen with age, leading to facial sagging and contour changes [192]. Aging skin also experiences decreased moisture content, leading to dryness, and in severe cases, itching, redness, and cracking erythema [193]. Abnormal pigmentation, another common feature of aging, can be attributed to an excess or deficiency of melanin [194].

Skin aging is also linked to cancer, primarily due to UV radiation, which damages cells, alters immune function, and causes oxidative stress and inflammatory reactions, leading to skin photoaging and skin cancer [195,196]. This is closely related to the p53 gene, which plays a role in DNA repair or the death of damaged cells [197]. However, mutations in the p53 gene, often caused by UV radiation, result in the dysregulation of apoptosis, expansion of mutated keratinocytes, generation of oxidative stress, immunosuppression, and induction of inflammatory responses, contributing to the development of melanoma and non-melanoma skin cancer [198]. Therefore, skin aging is not only externally visible but also causes various diseases and poses a health threat. Slowing down skin aging and reducing its negative effects significantly impact not only beauty but also the extension of a healthy lifespan [199].

The immune system of aging skin is more susceptible to viral infections. However, the specific mechanisms behind this decreased immune resistance are not fully understood. Kirchner et al. demonstrated that aged murine and human skin expressed reduced levels of antiviral proteins (AVPs) and circadian regulators, including BMAL1 and CLOCK [200]. The study discovered that BMAL1 and CLOCK play a role in controlling the rhythmic expression of AVP in the skin. The regulation of AVP by the circadian rhythm was diminished when the IL-27 signaling of immune cells was disrupted and when the BMAL1/CLOCK gene was deleted in mouse skin. Additionally, the knockdown of CLOCK using siRNA in human primary keratinocytes also reduced this circadian regulation. The results indicate that the regulation of cutaneous antiviral immunity is conserved throughout evolution and is sensitive to age-related changes in the circadian rhythm.

## 4. Cancer

Cancer is the second leading cause of death after CVD [201]. Breast cancer is the most common type, followed by prostate, bronchus and lung cancer, and colorectum (Table 3). Contributing factors to the increasing number of cancer deaths worldwide include a growing and aging population, exposure to chemicals or toxic compounds, and poor dietary habits [202]. According to the U.S. National Cancer Institute (NCI) definition, cancer is a group of diseases in which abnormal cells divide uncontrollably and spread to surrounding tissues, making cancer cell analysis crucial in studying the cells that cause cancer [203].

**Table 3:** Comparison of cancer incidence and mortality by cancer type between global and Republic of Korea, 2022.

	^1^ ASR Cancer Incidence per 100,000 Population		ASR Cancer Mortality per 100,000 Population	
Cancer Type/Site	Global	Republic of Korea	Global	Republic of Korea
Bladder	5.58	4.1	1.82	1.1
Brain, CNS	3.47	3.0	2.59	1.5
Breast	46.82	33.1	12.65	2.9
Cervix uteri	14.12	3.7	7.08	0.7
Colorectum	18.35	26.4	8.05	6.6
Corpus uteri	8.37	4.2	1.72	0.4
Esophagus	4.97	2.4	4.26	1.0
Gallbladder	1.15	6.6	0.83	3.9
Kidney	4.42	7.4	1.46	0.9
Leukemia	5.26	5.7	3.093	1.9
Lip and oral cavity	4.00	4.3	1.92	1.1
^2^ Liver	8.57	14.3	7.37	7.7
^3^ Lung	23.62	27.6	16.76	13.4
Multiple myeloma	1.79	1.8	1.11	0.7
Non-Hodgkin’s lymphoma	5.57	6.7	2.38	1.8
Ovary	6.65	3.7	3.97	1.3
Pancreas	4.69	7.7	4.21	5.6
Prostate	29.42	18.4	7.27	1.4
Stomach	9.18	24.0	6.09	4.1
Thyroid	9.12	47.6	0.44	0.2

^1^ ASR: age-standardized rates. ^2^ includes the liver and intrahepatic bile duct, ^3^ includes the lung and bronchus. Age-Standardized Rate (World) per 100,000, Incidence and Mortality, Both sexes, in 2022, Cancer Today, IARC.—https://gco.iarc.who.int (accessed on 7 June 2024).Global data were adapted from [204] with permission from Copyright © International Agency for Research on Cancer. Republic of Korea data were adapted from [205] (CC By 4.0) with permission from Copyright © 2022 Korean Cancer Association.

### 4.1. Breast Cancer

Breast cancer, a disease with high incidence in women, involves the proliferation of malignant cells in breast tissue, typically in the inner walls of the milk ducts or lobules that supply milk [206]. It is a metastatic disease with the potential to spread to various organs, making it challenging to treat [207]. The risk of developing breast cancer is closely tied to aging, with DNA methylation increasing as tissue ages [208]. The prevalence of the condition rises sharply until menopause and continues to increase gradually with age [209]. Notably, 80% of the patients are over the age of 50, and the number of deaths and diseases caused by this condition also increases [210,211]. 

Globally, breast cancer is the leading cause of death among women [203], and it is the first most common cancer in 2022 [204]. The incidence rate of breast cancer in Republic of Korea was 33.1 per 100,000 people, second only to thyroid cancer (Table 3) [205]. However, the mortality rate for breast cancer in Republic of Korea is relatively low, ranking sixth among all the cancer deaths compared to the global mortality rate, where it ranks second after lung cancer. 

Circadian disruption, such as altered sleep patterns and eating behaviors, can affect cancer outcomes in women with breast cancer [212]. The dysregulation of clock genes and hormone signaling, like estrogen and glucocorticoids, contributes to circadian desynchrony, affecting the expression of genes including Krüppel-like factor 9 involved in breast cancer development and progression [213]. Luminal A breast cancer disrupts the circadian clock, leading to irregularities in the rhythmic pathways and variable rhythmicity [214]. High rhythm strength in luminal A tumors is associated with lower 5-year survival rates, and larger tumors exhibit more synchronized rhythmicity in gene expression. Tumors with high rhythmicity intensity show a significant upregulation of genes associated with the epithelial/mesenchymal transition (EMT) pathway linked to cancer metastasis. In vitro studies have shown that reducing the rhythmicity of luminal A cells can slow down the rate of cell invasion [214]. 

The current standard of care for breast cancer includes surgical, radiation, and chemotherapy treatments. However, there are challenges [215]. In surgical intervention, local excision may not address the underlying disease, and patients may be dissatisfied with the cosmetic outcome. There are also cases where the ATM mutation occurs [216]. Radiation can be toxic to the heart, and chemotherapy can have side effects such as stopping the cell cycle or causing cell death [217]. 

### 4.2. Prostate Cancer

Prostate disease is closely associated with aging, a significant risk factor for disease development in numerous body systems, including the prostate. The prostate, in particular, is distinguished by an enlargement with age, in contrast to atrophy in other tissues [218]. Benign prostatic hyperplasia, a common condition characterized by the non-malignant enlargement of the prostate [219], is particularly prevalent in men over the age of 50, with a significant increase in prevalence with age [220]. It is known to cause lower urinary tract symptoms, including increased urinary frequency, urgency, decreased urine output, and nocturia [221,222]. The incidence of prostate cancer increases markedly with age. While the incidence of prostate cancer is 1 in 20,000 in men under the age of 39, it increases significantly to 1 in 7 in men aged 60–79 [223]. 

Adults with histologically normal tissues accumulate mutations as they age. These mutations can induce changes in otherwise healthy tissue, leading to genomic rearrangements and chromosomal replication abnormalities [224].

Recent studies have revealed an increase in the frequency of somatic mutations in cancer-related genes with age in normal adult tissues, indicating a process whereby normal cells evolve into precancerous cells, and occasionally into cancer cell clones [225,226]. Epigenetic profiling studies have demonstrated age-related changes in various human tissues, suggesting that genetic and epigenetic changes accumulate concurrently, contributing to aging [227,228].

Aging can also lead to circadian rhythm disruption, which is also associated with prostate cancer [229]. A reduction in the expression of circadian genes, including CLOCK, CRY1, CRY2, PER2, and BMAL1, has been associated with an increased risk of prostate cancer [230]. In particular, CRY1, CRY2, ROR, and BMAL1 have been implicated in the progression of prostate cancer [43,229]. PER1 may modulate prostate cancer risk through its role in regulating DNA damage and cell growth by interacting with proteins in cell cycle pathways. The other roles of PER1 include being regulated by androgens in the prostate, and the overexpression of PER1 can inhibit prostate cancer growth and induce cell death [231]. The disruption of circadian rhythms can affect hormone regulation, particularly the levels of androgens, which play a significant role in prostate cancer progression [232].

In patients diagnosed with prostate cancer, the disease is either confined to the prostate gland or is locally advanced. It can be effectively treated mainly by radiotherapy or hormonal therapy [233]. However, major treatments such as hormone therapy, radiation therapy, and surgery can cause serious side effects, including sexual dysfunction, urinary incontinence (surgery), and stool problems (radiation therapy) [234]. In light of these potential complications, natural compounds have gained significant attention in recent years due to their diverse anticancer effects [235,236]. 

### 4.3. Lung Cancer

In 2022, lung cancer had the highest mortality rate globally, despite being third in incidence after breast and prostate cancer [204] (Table 3). Risk factors include smoking, exposure to radon gas, asbestos, second-hand smoke, air pollution, genetic factors, and a history of asthma, pneumonia, and tuberculosis [237,238]. Common symptoms include cough and shortness of breath, with hemoptysis being the most specific [239]. However, symptoms often do not appear until the cancer has advanced, leading to late-stage diagnoses and poor prognosis [239]. 

Treatment options include surgery, radiation therapy, chemotherapy, and targeted therapy, with recommendations based on cancer type and stage [240]. Lung cancer is divided into two broad histologic classes: small-cell lung carcinomas (SCLCs) and non-small cell lung carcinomas (NSCLCs). NSCLC, which accounts for the majority of lung cancer cases, has shown promising results with vaccines [240] and targeted drugs like epidermal growth factor receptor (EGFR) inhibitors Erlotinib and Gefitinib [241]. 

Both viral and bacterial infections can lead to chronic inflammatory states that contribute to lung cancer development [242]. Anti-inflammatory treatment may reduce lung cancer incidence and mortality [243,244] but requires large-scale clinical trials to evaluate efficacy and safety [245]. COVID-19 severity is increased in individuals with pre-existing conditions like lung cancer due to the impact of the lung tumor microenvironment (TME) on viral infection [246]. Chronic inflammation is a key feature of chronic obstructive pulmonary disease (COPD), which is the third leading cause of death worldwide (Table 1 and Table 2). However, it is preventable and treatable. COPD is a potential contributor to the development of lung cancer. An understanding of the shared pathogenic pathways and the specific molecular and cellular mechanisms underlying COPD and lung cancer can provide valuable insights for future research to develop more effective strategies for the early diagnosis and treatment of lung cancer [247].

Several studies have demonstrated a strong correlation between immune dysfunction and the development of lung cancer. T cells and mesenchymal stem cells from lung cancer play a critical role in tumorigenesis and cancer progression, making them a key factor in lung cancer treatment [248].

Aging is possibly linked to a higher likelihood of developing lung cancer [249,250], primarily due to cellular DNA damage that accumulates over time and cumulative exposure to risk factors. A study using deep neural networks identified smoking frequency as a significant risk factor for lung cancer in men over 65 [251]. Quitting smoking sooner can reduce mortality from lung cancer later in life [237]. Proteomic changes in the aging lung mucosa suggest that neutrophils in the lungs of aging adults contribute to a dysregulated alveolar environment [252]. Studies often report stable or declining rates of lung cancer in elderly men, but increasing rates in women [253]. Biological factors like sex hormones and immune responses may contribute to these disparities in incidence and outcomes, although women generally exhibit better survival rates [254,255].

The disruption of circadian rhythms has significant implications for lung cancer. Studies show that circadian disruption (chronic jetlag) can lead to increased tumor burden in KRAS (Ki-ras2 Kirsten rat sarcoma viral oncogene homolog)-driven lung cancer mouse models, potentially through the upregulation of heat shock factor 1 target genes [256]. Circadian disruption is also associated with enhanced tumor growth due to the increased accumulation of immunosuppressive myeloid-derived suppressor cells within the tumor microenvironment in mice [257]. The involvement of circadian factors, such as the PER and CRY family’s genes in cancer signaling pathways, potentially impacts lung adenocarcinoma pathogenesis, suggesting that these could serve as novel biomarkers and therapeutic targets [258]. Inflammatory responses and oxidative stress are common factors leading to imbalanced autophagy and circadian rhythm dysfunction in COPD progression [259]. Autophagy impairment due to factors like cigarette smoke exposure can lead to cellular senescence and the increased secretion of inflammatory mediators in COPD patients. The disruption of circadian rhythms, as a consequence of inflammatory responses and oxidative stress, has a reverse effect on autophagy activity. These findings underscore the importance of maintaining healthy circadian rhythms for lung cancer prevention and treatment.

### 4.4. Colorectal Cancer

Colorectal cancer (CRC) is CRC is the fourth most prevalent and third deadliest cancer worldwide, with a higher incidence in developed nations and those with Westernized lifestyles [260,261].

However, recent trends show a global rise in cases, with Republic of Korea experiencing a significant surge in CRC incidence over the past few decades [262]. In 2018, Republic of Korea had the second-highest CRC incidence worldwide [263], making it a leading cause of mortality in the country, ranking third after lung and liver cancers [205] (Table 3). 

CRC encompasses malignancies arising in the colon or rectum, which may manifest as benign adenomas or progress into malignant adenocarcinomas [264]. Occasionally, premalignant adenomas may grow aberrantly, forming polyps or tumor-like structures in the colon or rectum, which may later evolve into cancerous masses [265]. 

The etiology of CRC primarily stems from Westernized dietary and lifestyle factors, such as the excessive consumption of red and processed meats and alcoholic beverages, inadequate intake of dietary fiber-rich foods, sedentary behavior, and obesity [266,267,268]. Environmental, genetic, and epigenetic influences also play significant roles in CRC development [269,270]. The etiology of CRC may vary based on anatomical subsites (e.g., proximal colon, distal colon, and rectum), with certain risk factors exhibiting stronger associations with specific subsites [271]. 

Recent studies have confirmed the importance of circadian rhythm in CRC occurrence and development [272]. The circadian rhythm regulator CLCOK can regulate CRC cells and contribute to the development of CRC: EMT through the activation of NF-kB, a known regulator of inflammatory response, and the RAS pathway, a key signaling pathway that controls various aspects of normal cell growth and malignant transformation [273]. Conversely, BMAL1 can reduce the incidence of CRC through its involvement in AKT, a serine/threonine kinase (also known as PKB) which is a central kinase that controls diverse processes including cell survival and apoptosis; the mammalian target of rapamycin (mTOR) which regulates cell proliferation; autophagy, metabolism, and apoptosis by participating in multiple signaling pathways; and p53 (tumor suppressor protein) by regulating various signaling pathways such as the Hippo pathway which regulates proliferation in intestinal stem cells [274,275]. Abnormalities in BMAL1 expression resulting from the disruption of the circadian rhythm can lead to the dysfunction of regulatory B cells in the intestinal epithelium and the apoptosis of CD4^+^ T cells [276], contributing to CRC development. 

CRC can be diagnosed at both early and advanced stages. Individuals with CRC may experience nonspecific symptoms such as unexplained weight loss, fatigue, abdominal pain or discomfort, iron deficiency anemia, and rectal bleeding [277].

### 4.5. Liver Cancer

The liver, the largest parenchymal organ in the human body, plays a crucial role in removing toxins and maintaining bioenergetics and cellular metabolism [278]. It comprises four lobes, each containing several lobules that lead to a common intrahepatic duct for bile drainage [279]. Irregular lifestyle patterns and the excessive use of substances like drugs or alcohol can stress the liver, leading to liver damage from factors like excessive fat accumulation, biotransformation metabolites, and bile stasis. Chronic stress can result in inflammation and degenerative liver injury, progressing to fibrosis, cirrhosis, and hepatocellular carcinoma [280,281] if untreated. Hepatocellular carcinoma (HCC), accounting for approximately 85% of the primary liver cancer cases, predominantly occurs in individuals with chronic liver disease [282]. The primary risk factors for HCC include long-term infection with hepatitis B virus (HBV) or hepatitis C virus (HCV), the consumption of foods contaminated with aflatoxin, excessive alcohol consumption, obesity, type 2 diabetes, and smoking [283]. Vaccination against HBV has significantly decreased the occurrence of HBV infection and the development of HCC in high-risk countries in Eastern Asia, making it a notable achievement in public health [283].

The circadian clock, recently suggested as a potential cause of liver cancer [272], is linked to obesity in people with irregular sleep patterns, such as night shift workers [284]. Circadian rhythm disruption can lead to damage to the sympathetic nervous system [285,286], bile stagnating, and overexpressed constitutive aldosterone receptors, accelerating liver cancer development cancer [287]. Disruption also increases the expression of the c-Myc oncogene, potentially leading to liver cancer through the decreased expression of tumor suppressor p53 [288]. 

In patients with chronic liver disease and a history of cirrhosis, HCC development involves hepatocyte necrosis, regeneration, and fibrous deposition due to inflammatory injury [282,289]. Early-stage liver disease can be treated with local surgical resection, liver transplantation, radiofrequency ablation, and transcatheter arterial chemoembolization [290]. However, most liver cancer patients miss the optimal timing for surgical treatment due to insidious onset, high malignancy, rapid development, and easy infiltration and metastasis [291]. For advanced HCC, the most common systemic drug therapy is tyrosine kinase inhibitors like sorafenib, lenvatinib, and regorafenib, but these often cause side effects that affect treatment effectiveness [292]. Immune checkpoint inhibitors have made a significant difference in tumor treatment, but the overall response rate in HCC patients is only 15–20% [293]. Therefore, research exploring new drugs or adjuvant therapies for the treatment of HCC is important.

### 4.6. Gastric Cancer

Gastric cancer, also known as stomach cancer, is one of the most prevalent and life-threatening tumors worldwide, despite a steady decline in incidence rates [294]. It is more common in males and has the highest incidence and mortality rates in East Asia [295]. In the United States, it is more common in Hispanic Americans, African Americans, Native Americans, Asian Americans, and Pacific Islanders than in non-Hispanic White people [296]. The primary risk factor for gastric cancer and gastric non-Hodgkin’s lymphoma is *Helicobacter pylori* (*H. pylori*) infection [297,298]. Other risk factors including obesity and gastroesophageal reflux disease, are the main risk factors for gastric cancer, especially in younger generations, indicating lifestyle and health conditions significantly influence risk [299]. Salty food [300], smoking [301], and alcohol consumption are also risk factors [302]. The rising prevalence of autoimmune gastritis, changes in the gastric microbiome, and increased use of antibiotics among younger generations are also suspected to contribute to the increasing incidence of early-onset gastric cancer [299,303]. Age is an independent risk factor for gastric cancer-related mortality, with its impact differing depending on the stage of the cancer [304]. Younger patients have better survival rates, while older patients showed a higher frequency of tumors with microsatellite instability and ARID1A (AT-rich interactive domain 1A gene) mutations [305]. Researchers are exploring novel methods for diagnosing stomach cancer by discovering biomarkers. They have developed a prognostic risk model based on multi-omics data, cellular aging-related lncRNAs, and immune cell counts to facilitate the early diagnosis and prognosis of gastric cancer [306,307,308]. Gastrointestinal metaplasia (GIM) is a precancerous change that increases the risk of dysplasia and gastric cancer [309,310]. The presence of GIM increases with age, as observed in the routine gastric examinations of Koreans [311], suggesting that chronic inflammation caused by *H. pylori* infection may contribute to the progression of precancerous GIM with age, potentially leading to gastric cancer. Regular endoscopic surveillance for GIM is necessary to identify it in its early stages and develop preventive strategies against the progression of gastric cancer [310]. 

The disruption of circadian rhythms plays a significant role in gastric cancer progression and treatment outcomes [312]. The mRNA expression of both nuclear receptor subfamily 1 group D member 1 (NR1D1) and nuclear receptor subfamily 1 group D member 2 (NR1D2) is elevated in gastric cancer compared to normal tissue [313]. NR1D1 and NR1D2 are transcription factors that bind ROREs and act as transcriptional repressors to inhibit the expression of BMAL1:CLOCK [314], underlining the importance of considering circadian rhythms in gastric cancer development and treatment [313]. Specifically, in human epidermal growth factor receptor 2 (HER2)-positive advanced gastric cancer, the circadian oscillation of glycolysis controlled by hexokinase 2 (HK2) and PER1 contributes to trastuzumab resistance, which is an inevitable major problem in chemotherapy targeting HER2 using that drug, whereas Metformin-based chronotherapy disrupts the BMAL1-CLOCK-PER1-HK2 axis, affecting glycolysis oscillation to overcome trastuzumab resistance [315]. 

### 4.7. Pancreatic Cancer

Pancreatic cancer, typically diagnosed in older adults, is a high-risk cancer due to its lack of significant symptoms and difficulty in early detection [316]. About 90% of the newly diagnosed patients are over 55 years old. Despite its complex nature and generally unknown exact causes, several risk factors have been identified, including smoking, obesity, excessive alcohol consumption, exposure to certain chemicals, family history, and genetic mutations [317]. Heavy drinking can lead to chronic pancreatitis, increasing the risk of pancreatic cancer. People with diabetes, especially T2DM patients, who have a 53% elevated mortality rate when diagnosed with pancreatic cancer, are also at increased risk [122]. The KRAS protein, which aids cell growth and movement, can cause cancer when it changes. KRAS mutations typically occur early in the development of pancreas ductal adenocarcinoma (PDAC), and are present in 90–95% of PDAC cases [318]. An understanding of PDAC pathogenesis has led to the development of the first drug candidates capable of targeting the key oncogene KRAS in PDAC [318,319]. These candidates have shown clinical success in inhibiting KRAS in lung cancer as well as in preclinical-stage pancreatic cancer [319].

A study using a machine learning method called cyclic ordering by periodic structure (CYCLOPS) revealed an attenuated biological clock in pancreatic cancer tissue compared to normal tissue [320]. This dysfunctional clock leads to accelerated cancer growth, worse survival, chemoresistance, and the enrichment of cancer-related pathways [320]. The disruption of the circadian rhythm in mice, either by deleting the *Bmal1* gene or through chronic jet lag, exacerbates fibrotic phenotype in tumors, promoting cancer metastasis in pancreatic cancer, involving cancer-associated fibroblasts and transforming growth factor (TGF-β) signaling pathway [321,322].

## 5. Natural Compounds in Aging and Cancer Therapy and Prevention

Phytochemicals, which are chemical components found in plants, have been shown to have a wide range of bioactive and therapeutic potentials. The major active compounds are phytosterols, polyphenols, flavonoids, terpenoids, saponins, alkaloids, carotenoids, aromatic acids, organic acids, essential oils, and protease inhibitors [323,324]. Major plant ingredients used for medical purposes are listed in Table 4. 

### 5.1. Natural Compounds in Age-Related Diseases

#### 5.1.1. Cardiovascular Diseases (CVDs)

Oxidative stress, inflammation, and apoptosis are crucial for maintaining cellular function. However, when not properly regulated, they can result in tissue damage and cell death, ultimately contributing to CVDs [335]. Natural compounds act by reducing oxidative stress, inflammation, and cell death, which play a role in CVDs. They offer antioxidant effects, prevent disease progression, and promote angiogenesis for treatment efficacy.

Saponins exhibit cardioprotective effects by offering antihypertensive and antiatherosclerotic activities, making them valuable in treating CVDs like myocardial infarction and coronary heart disease [336]. Sleep deprivation has been linked to cardiovascular complications, including arrhythmias and myocardial damage [337]. However, saponins in ginseng roots have been shown to protect the heart by improving blood flow and resolving heart problems in sleep-deprived mice. The cell signaling pathway that is activated by saponins is phosphatidylinositol 3-kinase(PI3K)/AKT/mTOR, an intracellular signaling pathway important in regulating the cell cycle. This pathway prevents excessive cell breakdown and death, thereby preventing sleep deprivation-induced heart damage. The use of natural compounds in botanical drugs offers a promising therapeutic benefit for atherosclerosis and related CVDs. This is achieved by targeting the mTOR signaling pathway, which regulates a number of processes, including the immune response, autophagy, cellular senescence, and lipid metabolism [338]. Representative natural compounds that target the mTOR signaling pathway include curcumin, resveratrol, epigallocatechin-3-gallate (EGCG), quercetin, rapamycin, and berberine. 

Curcumin, also known as diferuloylmethane, is the primary natural polyphenol present in the rhizome of *Curcuma longa* (turmeric) and other *Curcuma* spp. and has been found to selectively interact with various signaling molecules and exhibit cellular effects, thereby contributing to its diverse range of health benefits [339]. Resveratrol, also known as 3,5,4′-trihydroxy-trans-stilbene, is a compound that falls under the group of polyphenols called stilbenoids [340]. It consists of two phenol rings that are connected to each other by an ethylene bridge. This has been identified in over 70 different plant species, particularly in the skin and seeds of grapes. Resveratrol exhibits a diverse array of biological characteristics, including antioxidant, cardioprotective, neuroprotective, anti-inflammatory, and anticancer properties [340]. EGCG is the most abundant and biologically active polyphenol in green tea extract. In several animal models, EGCG’s antioxidant, anti-inflammatory, and lipid-modulating effects protect against atherogenesis. Thus, EGCG reduces vascular inflammation, foam cell formation, and smooth muscle cell apoptosis. EGCG has been shown to protect against cardiac hypertrophy [341]. Quercetin, a natural flavonoid, is abundant in fruits, onions, tea, and red wine. Quercetin has many CVD medicinal properties, including antioxidation, antiplatelet aggregation, myocardial fibrosis reduction, ventricular remodeling and cardiac function improvement, vascular endothelium protection, anti-arrhythmia, anti-heart failure, ischemia/reperfusion injury prevention, and blood pressure regulation [342]. Berberine is an alkaloid extracted from some plants including species of *Berberis*. It has a wide range of pharmacological effects, including anti-diabetic, antihypertensive, antidepressant, and anticancer effects [343]. Berberine has been shown to treat cardiac hypertrophy, HF, atherosclerosis, and stroke. The efficacy of it in treating multiple diseases is due to its multi-target pharmacological profile, which improves AMP-activated protein kinase (AMPK), the human protein tyrosine phosphatase 1B, the silent information regulator of transcription1 (SIRT-1), the proprotein convertase subtilisin/kexin type 9, the low-density lipoprotein receptor, the peroxisome proliferator-activated receptor (PPAR), the nuclear factor kappa-light-chain-enhancer of activated B cells (NF-κB), and gut microbiota [344].

The nuclear factor erythroid 2-related factor 2 (Nrf2) is a transcription factor that plays a pivotal role in regulating the expression of antioxidant and cytoprotective enzymes in response to oxidative stress [345]. It has been demonstrated that compounds that activate Nrf2 signaling have the potential to treat CVD by regulating antioxidant enzymes and inhibiting inflammation through the Nrf2 and NF-kB pathways [345]. Bacosides, silymarin, withanolin, ECGC, and curcumin are representative examples of natural Nrf2 activators that have been demonstrated to reduce oxidative stress and enhance the expression of antioxidant enzymes, including superoxide dismutase (SOD) and catalase [346].

Phenolic compounds, which are well-known for their antioxidant properties, have emerged as potential therapeutics for CVD by modulating biological rhythms, which significantly influence CVD risk factors [347]. Pharmacological agents targeting the molecular clock protein PER2 have shown promise in treating myocardial ischemia, highlighting the importance of manipulating the molecular clock for cardiovascular health [3]. Moricizine has been identified as a clock-period lengthening compound, demonstrating novel clock-modulating activities that could be beneficial in combating heart diseases [348]. Several studies have shown that phenolic compounds have different effects depending on the time of administration. Resveratrol has also been shown to modulate the expression of clock genes such as SIRT-1, PER1, PER2, BMAL1, and REV-ERBα in several animal studies [349]. Quercetin is a SIRT-1 activator [350] and an antioxidant, and has vasodilatory effects through endothelial nitric oxide synthase (eNOS) activation [351], reduces blood pressure by inhibiting angiotensin-converting enzyme [352], and regulates the body clock [353].

#### 5.1.2. Degenerative Brain Disorders

Curcumin has shown positive effects on brain diseases. In AD, curcumin can reduce amyloid β production by modulating pAKT activation, PI3K/AKT, and NF-κB signaling [354]. It also reduces neurotoxicity and increases neurogenesis in the brain through modulation of WNT levels, T-cell factor/lymphoid enhancer factor transcription factors, and cyclin-D1 activity [355]. In PD, curcumin promotes autophagy through interference with alpha-synuclein aggregation and stimulates proteins like LC3-II which is a standard marker for autophagosomes, transcription factor EB which is a key nuclear transcription factor in control of autophagy, and lysosomal-associated membrane protein 2 isoform A (LAMP2A) which is the key component of chaperone-mediated autophagy to induce neuroprotection and prevention [356]. Berberine and resveratrol promote autophagy activity through the SIRT1-dependent pathway [357].

Berberine has demonstrated therapeutic effects on neurodegenerative diseases [231]. It modulates AKT/glycogen synthase kinase-3 beta (GSK-3β)/extracellular signal-regulated kinases 1/2, survival/apoptotic signaling, and inhibits c-Jun N-terminal kinases (JNK) and caspase-3 activity [98,358]. In AD, berberine may delay or prevent the onset by reducing the production of Aβ [98,358]. It suppresses the synthesis of interleukin-6 and monocyte chemotactic protein-1 stimulated by Aβ and downregulates the expression of cyclooxygenase 2 (COX-2) and inducible nitric oxide synthase (iNOS) by blocking certain signaling pathways [359,360].

The efficacy of resveratrol in the treatment and prevention of AD is being studied [361]. It affects Aβ peptides and Tau protein, which are significant factors in the pathogenesis of brain disease [362]. The increased expression of SIRT-1 may be neuroprotective by reducing levels of pro-inflammatory cytokines through reducing NF-κB/IL-1β/NLRP3 (NOD-, LRR- and pyrin domain-containing protein 3) signaling [363]. Alternatively, increased SIRT-1 may reduce amyloidogenic processing through β-site amyloid precursor protein cleaving enzyme reduction [364] and act in concert with increased AMPK to increase the expression of PPAR-gamma coactivator-1α, which may protect against the development of brain disease through increased mitochondrial biogenesis [365].

EGCG, found mainly in green tea, exhibits potent antioxidant and anti-inflammatory effects and has shown protective effects against AD [366]. EGCG prevents Aβ-induced hippocampal neuronal cell death by acting as an antioxidant. It also reduces Aβ accumulation, improves cognitive function, and has neuroprotective effects in AD models [367]. Additionally, nano-EGCG has been shown to reverse AD hallmarks and benefit cortical and hippocampal neurons [368]. EGCG inhibits oxidative stress, reduces neuron apoptosis, and inhibits tau aggregation, thereby modifying tau’s three-dimensional structure [369].

Nobiletin is a natural compound that has shown promise in targeting circadian rhythms to treat degenerative brain disorders like AD [370]. Nobiletin targets circadian rhythms to mitigate neuroinflammation and disease hallmarks in AD, showing potential for treating degenerative brain disorders [371]. Research indicates that nobiletin can directly activate circadian cellular oscillators, improve metabolic health, and promote healthy aging in disease models and naturally aged mice [370]. In AD model mice, nobiletin treatment reduced amyloid burden, improved cognitive function, and modulated clock and clock-controlled gene expression in the cortex [370].

Melatonin administration has been linked to improvements in circadian rhythms, nighttime sleep, daytime wakefulness, and rest/activity rhythms in patients with neurodegenerative diseases [372]. Melatonin administration, especially in combination with bright light therapy, has shown promise in enhancing treatment outcomes by targeting circadian clock mechanisms in neurodegenerative diseases [373]. Resveratrol, 1,8-cineole, and the herbal extract of Ashwagandha target circadian rhythms for treating degenerative brain disorders [374,375].

#### 5.1.3. Diabetes Mellitus

Tannat grape variety is suitable for wine production and contains high concentrations of quercetin. The extract prepared using methanol exhibited a considerable amount of quercetin. Quercetin has antioxidant, anti-diabetic, anti-obesity, and anti-inflammatory properties. The consumption of Tannat grape skins has been demonstrated to significantly reduce the risk of developing diabetes mellitus [376]. The extract retains high antioxidant activity even after digestion and helps to regulate blood sugar levels by inhibiting the key digestive enzymes α-glucosidase and α-amylase.

Berberine has been noted for its ability to stimulate insulin secretion without causing hypoglycemic side effects, such as those observed with insulin secretagogues used to treat T2DM [377]. Specifically, it reduced glycosylated hemoglobin, the percentage of hemoglobin bound to blood glucose, by up to 0.75 compared to the placebo control [378]. Furthermore, it was also effective in accelerating the repair and wound healing of diabetic ulcers, a common complication of diabetes [379].

Silymarin has been reported to have various beneficial effects, including hepatoprotective, cardiovascular protective, anti-diabetic, anti-inflammatory, antioxidant, and anticancer properties. However, the specific mechanisms underlying these effects are not yet fully understood. Silymarins from *Silybum marianum* have been identified as natural compounds that target the circadian clock by disrupting the CRY1-CLOCK interaction, enhancing circadian rhythm amplitudes [380]. Silymarin and silybin A could be utilized in the development of treatments for diseases associated with circadian rhythm disruptions, such as chronic diseases. Additionally, the anti-parasitic drug ivermectin has been found to restore insulin secretion by activating the human purinergic G protein-coupled receptor P2Y1, a clock-controlled mediator, in circadian mutant beta cells [381]. AKT activators, such as SC79, have been shown to shorten the circadian period and advance the phase [382], indicating their ability to modulate the circadian rhythm. AKT activators can serve as potential modulators of the circadian clock.

Melatonin is a naturally occurring compound that plays a key role in regulating the circadian rhythm. Research has shown its potential in treating diabetes by regulating insulin secretion, improving glucose homeostasis, and acting as a cytoprotective antioxidant [383]. Melatonin has been found to improve the impairment of Leydig cell steroidogenic function caused by diabetic hyperglycemia by activating the SIRT-1 pathway [384].

#### 5.1.4. Musculoskeletal Disorders

Arthritis medications can be categorized into several types, including NSAIDs, selective COX-2 inhibitors, narcotic analgesics, and topical analgesics [385,386]. Additionally, anti-tumor necrosis factor (TNF) agents and antibody therapies have been used to treat RA by reducing inflammation, pain, and joint damage [387]. OA is a degenerative disease characterized by the gradual breakdown of cartilage and surrounding tissues in the joints, while RA is an autoimmune disease where the body’s immune system mistakenly attacks its own organs or tissues, leading to inflammation. Anti-inflammatory treatments are commonly used for both conditions. ROS have been linked to the development of OA [388], and antioxidants are known to play a crucial role in its management. Phytochemicals derived from medicinal plants have shown potent anti-inflammatory properties by regulating molecular mechanisms to alleviate inflammation-associated diseases [389]. Escin, quercetin, and capsaicin are phytochemicals with anti-inflammatory properties that can be used to treat arthritis [389]. Plants from African forests contain phytochemicals like quercetin, rutin, catechin, and kaempferol, known for their anti-inflammatory properties, and potentially beneficial in treating arthritis, particularly RA [390]. Natural compounds have been shown to inhibit oxidative stress signaling in OA, suppress inflammation-related gene expression (prostaglandin E2, iNOS, and COX-2), and inhibit the expression and protein levels of MMP3, MMP13, and ADAM metallopeptidase with thrombospondin type 1 motif 4 induced by inflammatory cytokines such as IL-1β, IL-6, or TNF-α [391]. Furthermore, chondrocytes treated with antioxidant phytochemicals or their plant extracts showed upregulated levels of catalase, SOD, glutathione peroxidase, and reduced glutathione, indicating that the natural compounds used have antioxidant effects [392].

Phytochemicals like andrographolide, berberine, curcumin, ginsenoside, hesperidin, kirenol, madecassocide, and other polyphenolic compounds exhibit anti-inflammatory properties and are potential treatments for arthritis [393].

Current drug treatments for OP, such as bisphosphonates, selective estrogen receptor (ER) modulators, parathyroid hormone peptides, monoclonal antibodies, and estrogen, have been effective in reducing the risk of fracture in patients with OP [394]. In addition, natural compounds such as phytoestrogens, antioxidants, and multimodal therapeutic agents show potential in the fight against OP.

Phytoestrogens like isoflavones mimic estrogen, can bind to ERs, inhibit bone resorption by inhibiting osteoclast differentiation, enhance bone formation, and increase bone mineral density, potentially treating OP in postmenopausal individuals. Isoflavones like genistein and daidzein found in soy products can affect bone metabolism and mechanical strength by acting on osteoblasts and osteoclasts. Lignans and coumestans, including isoflavones, are the three main components of phytoestrogens [395].

ER-α and ER-β are transcription factors that play a role in regulating numerous physiological processes, and their dysfunctional signaling can contribute to the development of various diseases, such as cancer, metabolic and CVDs, neurodegeneration, inflammation, and OP [396]. Terpenoids have agonistic and/or antagonistic activities for ER-α and ER-β [395].

Sulforaphane, found in broccoli sprouts, cabbage, kale, and cauliflower, effectively suppressed the differentiation of osteoclasts by inhibiting RANKL-mediated reactive oxygen species (ROS) production through its antioxidant properties [395]. A review of the experimental and cohort studies of the effects of lycopene on bone loss in elderly women and laboratory animals suggests that lycopene may contribute to reducing oxidative stress caused by decreased estrogen secretion [397]. Polysaccharides from *Agrimonia pilosa* and its derivatives demonstrated anti-apoptotic effects in osteoblasts, indicating a potential role in preventing OP-related cell apoptosis [398].

Traditional plant flavonoids like quercetin, icariin, hesperitin, naringin, chrysin, and pueraria have been explored for their potential skeletal protective effects and as potential alternative treatments for OP [399].

Resveratrol exhibits anti-osteoporotic effects by activating SIRT-1, a protein that helps protect cells from oxidative stress and supports bone health by improving mitochondrial function, reducing bone loss [400], and inhibiting p53 [401]. Resveratrol activates the AKT/mTOR signaling pathway, which plays a significant role in regulating autophagy which is a process that helps bone cells remove damaged components and function better, and cell survival, thereby contributing to better bone health in osteoporotic conditions [400].

Curcumin has potential in maintaining bone health in different OP models and has gained significant research interest [402]. Curcumin blocked osteoclast differentiation [403], promoted osteoblast proliferation [404], and activated the WNT/β-catenin pathway, leading to exerting osteoprotective effects in glucocorticoid-induced OP [405] and ovariectomized OP rat [406].

Curcumin also has shown benefits in preventing muscle degeneration, maintaining satellite cell function, protecting mitochondrial health, and reducing inflammation and oxidative stress [407]. In addition to curcumin, natural compounds such as resveratrol, catechins, soy protein, and ginseng have been demonstrated to stimulate anabolic factors, including myogenin, Myf5, and MyoD, which are essential for muscle growth and regeneration [408]. Pinoresinol and vanillic acid, extracted from *Catalpa bignonioides* Walt, have been found to stimulate muscle cell proliferation through the insulin-like growth factor-1 receptor (IGF-1R)/AKT/mTOR pathway, potentially alleviating sarcopenia [409,410]. Additionally, phytochemicals like magnolol, fisetin, and sclareol have shown promise in ameliorating muscle atrophy induced by cisplatin through inhibiting IL-6 and myostatin expression and improving root canal diameter [411]. The 5,7-dimethoxyflavone found in *Kaempferia parviflora* and extra virgin olive oil has shown promise in slowing muscle wasting and sarcopenia [412,413]. Natural compounds, including resveratrol, quercetin, ursolic acid, ecdysone, and vitamin D, have been studied for their potential in regulating skeletal muscle health [414]. These compounds, often referred to as “plant exercise pills”, mimic sex hormones and offer protective and regulatory effects on skeletal muscle, potentially mitigating issues like muscle atrophy, the loss of mass, and metabolic disorders.

Disruptions in circadian rhythms have been linked to OP, highlighting the importance of maintaining circadian balance for musculoskeletal health. Aligning meal times with the body’s circadian rhythm can enhance nutrient absorption and utilization, benefiting the musculoskeletal system [35]. Adequate protein intake is crucial for muscle repair, growth, and maintenance. Vitamin D is essential for bone health and calcium absorption, making it valuable for preventing musculoskeletal conditions like OP. Additionally, incorporating the sources of omega-3 fatty acids into the diet can reduce inflammation and promote joint health, providing added benefits to the musculoskeletal system.

Melatonin exerts a variety of physiological effects, including inducing anti-inflammatory and antioxidant functions and resetting circadian rhythms, and is also involved in the maintenance of bone and cartilage health [415].

#### 5.1.5. Ophthalmology Disorder

Oxidative stress plays a significant role in various eye pathologies such as glaucoma, cataracts, and age-related macular degeneration [416,417]. Compounds like baicalein, forskolin, cannabinoids, ginsenoside, resveratrol, and hesperidin have shown effectiveness in lowering intraocular pressure, a key factor in glaucoma treatment, and in exhibiting antioxidant, anti-inflammatory, and anti-apoptotic mechanisms [416]. Additionally, polyphenols from *Citrus bergamia*, luteolin, curcumin, coenzyme Q10, grape seed extract, and polyunsaturated fatty acids have demonstrated antioxidant and anti-inflammatory properties, aiding in eye protection and potentially benefiting the early stages of eye disorders, including glaucoma [417]. Furthermore, natural compounds like thymoquinone, catechin, EGCG, and quercetin are being explored using nanotechnology-based delivery systems to enhance bioavailability for ocular applications, addressing anatomical and physiological barriers in ocular drug delivery [418].

Melatonin, which is synthesized in the eye, has been identified as a promising treatment for eye diseases due to its ability to reduce free radicals and inflammation. This makes it a suitable candidate for age-related eye diseases and diabetic retinopathy [419]. In animal models, melatonin has been shown to reduce cataract formation by decreasing lipid peroxidation and enhancing antioxidant activity, suggesting its potential in preventing cataracts. Melatonin can help regulate intraocular pressure (IOP), which is crucial for managing glaucoma, as studies suggest it can significantly reduce IOP and may be involved in the circadian rhythm of IOP [420]. Melatonin’s ability to improve sleep disorders can be beneficial for treating dry-eye symptoms, as better sleep can alleviate these symptoms and improve overall eye health. Studies have demonstrated that melatonin protects ocular tissue, making it an effective treatment for age-related eye diseases when used alone or in combination with other medications.

#### 5.1.6. Skin Aging

Efforts to treat skin aging have continued, including the use of oral vitamin intake, hormonal treatment, and management through cosmetics. However, due to the potential for adverse effects and the presence of chemicals and toxins, the use of common cosmetics and treatments has been limited [421], especially for aged skin [422]. Considering this necessity, the utilization of compounds derived from natural sources has emerged as a more efficacious and dependable approach [423].

Skin aging is primarily characterized by a reduction in collagen content, thinning of the skin, and the development of wrinkles and dryness due to a loss of moisture [193,424]. Additionally, the expression of MMPs, which reduce and degrade ECM proteins, is increased. Various natural extracts have been studied for their potential to reduce these symptoms and improve the signs of aging [425].

For instance, the extract of the young leaves of evening primrose (*Oenothera biennis*) has been demonstrated to moisturize the skin and improve wrinkles [426]. The resulting extract using 50% ethanol contained the highest concentration of gallic acid, with a total of 28.28 mg/g. Gallic acid is renowned for its multifaceted effects on the skin, exhibiting potent antioxidant and free radical scavenging capabilities, which protect cells and organs. Due to the complex composition of evening primrose extract, which includes gallic acid, it is capable of targeting and improving MMP-1, which decomposes elastase and collagen. Furthermore, it enhances skin hydration by promoting collagen synthesis and increasing the expression of related genes.

*Stenocarpus sinuatus*, an Australian tree belonging to the Proteaceae family, has been found to exhibit anti-collagenase, anti-elastase, anti-tyrosinase, and anti-hyaluronidase activities from the leaf extract using the hexane-soluble fraction of a *Stenocarpus sinuatus* leaf methanol extract which contains α-tocopherol, γ-sitosterol, neophytadiene, and β-tocopherol, making it a promising agent for the treatment of aging skin [427]. The main constituent of the extract has been determined to be vitamin E (α-tocopherol), making up 52.59% of the extract. The extract’s ability to combat aging can be ascribed to its abundant vitamin E content, as well as the combined effects of other volatile components.

*Ocimum sanctum* Linn, also known as the “Queen of Herbs”, has been demonstrated to delay the aging of the skin by inhibiting the breakdown of collagen, a key extracellular matrix protein, through the inhibition of MMP-1 by up to 80% [428]. The extract was prepared with ethanol. The principal peak of this extract corresponds to rosmarinic acid, which was identified as the predominant component. Rosmarinic acid is a type of phenolic acid, a phenolic compound that has been demonstrated to inhibit and kill tumor cell proliferation, as well as reduce metastasis and inflammation. These properties render rosmarinic acid an efficacious agent against the processes of aging and carcinogenesis. The extract exhibits a higher inhibitory activity than pure compounds such as ursolic acid, linalool, and rosmarinic acid.

Sargassum is a brown alga that is found in tropical and subtropical areas in shallow marine meadows [429]. It contains polysaccharides with biologically active components like polyphenols, steroids, and terpenoids. These polysaccharides are widely used in cosmetics for their anti-inflammatory, anticoagulant, and antiviral properties, offering skin protection. Sargassum has been observed to have positive effects on wrinkle improvement and skin whitening, with up to 64.72% inhibition of tyrosinase and 30.12% inhibition of elastase activity [430].

Grapes, particularly their skins, are renowned for their beneficial effects, which are attributed to a compound known as resveratrol [431,432]. Various extracts have shown effectiveness in preventing and improving the effects of UV radiation on the skin. The ethanol treatment and freeze-drying methods employed in the study yielded high concentrations of resveratrol, as confirmed by subsequent analysis [433]. This compound engages the Nrf2/ heme oxygenase-1(HO-1) signaling pathway, thereby providing antioxidant activity that protects the skin from oxidative damage. This resulted in an enhanced nuclear level of Nrf2, which was previously downregulated following UVB irradiation. Furthermore, the findings indicate that resveratrol enhances the cytoplasmic level of HO-1, a major downstream antioxidant enzyme. This suggests that resveratrol plays a role in the Nrf2/HO-1 signaling pathway. These results are due to the mechanism by which Nrf2, a major regulator of antioxidant enzymes, is released from the Nrf2-Keap1 complex, moves to the nucleus, and binds to the promoter region of the inducible antioxidant enzyme gene. This involvement allows resveratrol to reduce the formation of wrinkles and the increased thickness of the epidermis which is a consequence of photoaging.

*Adenocaulon himalaicum* Edgew. (AHE), a plant native to Korea’s Ulleungdo Island, has been used in traditional medicine and shown to have various effects on skin photoaging cells [434]. AHE extracts were obtained with ethanol solvents. Research has demonstrated their ability to reduce UV-induced cytotoxicity, inhibit collagen degradation, decrease MMP-1 production via the mitogen-activated protein kinases/activator protein-1 (MAPK/AP-1) signaling pathway, and enhance procollagen type 1 protein production. The bioactive component of the AHE extract has been identified as neochlorogenic acid. It has been confirmed that neochlorogenic acid modulates the MAPK/AP-1 signaling pathway and skin moisturizing factors in a manner similar to that of the AHE extract. These findings collectively suggest that both the AHE extract and neochlorogenic acid hold promise as potential treatments for photo-aging skin conditions.

Safranal, a monoterpene aldehyde found in iris and saffron flowers, has several biological functions, including antioxidant activity and anti-enzyme properties related to skin aging [435]. It exhibited superior anti-elastase activity compared to plant extracts from *Areca catechu*, *Cinnamonum cassia*, *Myristica fragrans*, *Curcuma longa*, *Alpiniakatsumadai,* and *Dryopteris cassirrhizoma*, all of which contain anti-aging compounds [436].

Curcumin, the principal constituent of turmeric, has anti-inflammatory and antioxidant effects [437]. UV radiation exposure is a significant contributor to the deterioration of the skin, resulting in the development of fine lines, wrinkles, and other visible signs of aging. A recent study has confirmed that the solvent extracts from the rhizome of *Curcuma longa* contain significant amounts of arturmerone. Arturmerone essential oil has been shown to suppress the symptoms of photoaging skin and reduce inflammatory responses [438].

Green tea, notable for its high catechin content, has been used in various biological contexts [439]. Catechin is a flavonoid antioxidant that has a significant impact on antioxidant activity [440]. Green tea extract has garnered attention for its diverse array of anticancer and antioxidant biological properties, attributed to its numerous constituents, including polyphenols, caffeine, and theanine [441]. Research has shown that green tea (*Camellia sinensis*) leaf extract can restore the expression of UV-damaged metalloproteinase inhibitor 3 (TIMP3) in the skin, which helps control inflammation and promote skin regeneration [442]. TIMP3 exhibits circadian expression in the skin and regulates the circadian machinery. It acts in a manner analogous to PER1, a known core clock gene, and the epidermal circadian regulation gene Aquaporin 3. Dutch-based green tea extract upregulated TIMP3 by up to 22.9% at a non-toxic concentration, suggesting its potential to restore the circadian expression of TIMP3 disrupted by UV irradiation (UVR). Compared to the standard 70% ethanol extract, the Dutch and new green solvent (NGS) extracts were found to be rich in amino acids and catechins, respectively. Interestingly, co-treatment with Dutch and NGS extracts resulted in a remarkable increase of over 200% in TIMP3 expression under UVR. These findings suggest that the introduction of different components through co-treatment may play a significant role in enhancing TIMP3 recovery under UVR conditions.

The immune system of aging skin is more susceptible to viral infections. According to Kirchner et al., a reduction in the expression levels of the antiviral protein (AVP) and its circadian regulators BMAL1 and CLOCK in the skin of both aged mice and humans was observed [200]. The regulation of AVP by the circadian rhythm was diminished when the BMAL1/CLOCK gene was deleted in mouse skin. The administration of the circadian enhancers nobiletin and SR8278 was discovered to decrease the occurrence of herpes simplex virus 1 infection in epidermal explants and human keratinocytes, relying on the presence of the BMAL1/CLOCK protein complex. Moreover, the application of circadian enhancement treatment was found to counteract the vulnerability of aged mouse skin and human primary keratinocytes to viral infection. The results emphasize the importance of circadian restoration as a potential way to combat viral infections in older populations.

### 5.2. Natural Compounds in Cancer Treatment

#### 5.2.1. Breast Cancer

Given the significant adverse effects associated with hormone replacement therapy, including CVD and breast cancer, the utilization of compounds derived from natural sources has emerged as a more efficacious and dependable approach. Indeed, there is a paucity of evidence to substantiate the efficacy of vitamin intake, while hormone replacement therapy is associated with significant adverse effects, including CVD and breast cancer [443].

Curcumin has been demonstrated to inhibit the invasion and metastasis of triple-negative breast cancer in the absence of positive signs such as ER, progesterone receptor (PR), and HER2. These three tumors are highly invasive and present significant challenges in terms of treatment. It is a challenging disease to diagnose and treat, accounting for 15–20% of all breast cancers and exhibiting a low survival rate [444,445,446]. In response, curcumin inhibited this process, reducing tumor invasiveness and preventing metastasis. The inhibitory effect of curcumin is greater than that of DMSO, which is toxic to human breast cancer cells. This effect inhibits cell colony formation and cell invasion and migration. Additionally, curcumin inhibited the formation of mammospheres, which regulate intratumoral cell movement, interaction, and growth within cancer tissues. Following the treatment and culturing of 10,000 mammospheres with curcumin, fewer than 50 were observed on the seventh day, thereby confirming the effectiveness of curcumin in inhibiting the formation of these structures.

Quercetin has been demonstrated to prevent breast cancer metastasis, a process that is promoted by circadian rhythm disruption [447,448]. Quercetin has been demonstrated to inhibit the invasion of human breast cancer cells and to inhibit the proliferation of these cells. This suppresses cancer metastasis by inhibiting cancer cell migration, and this effect was expressed even when the circadian rhythm was disturbed. Light/dark shift, one of the circadian rhythm disturbance factors, increased lymph nodes and increased the neutrophil/lymphocyte ratio. It also represents an indicator of inflammation and a prognostic factor for breast cancer. Furthermore, given that chronic inflammation is associated with cancer, malignancy, and a poor prognosis, light/dark shifts and night shifts also affect cancer metastasis and prognosis. It significantly inhibited lymph node metastasis, particularly metastasis to the iliac and renal lymph nodes. These results confirm that quercetin prevents breast cancer lymph node metastasis promoted by the disruption of the circadian rhythm.

Berberine demonstrated anticancer properties by inhibiting the survival of triple-negative breast cancer cells that express ER, PR, and HER2 [449]. This process not only involved the simple apoptosis of cells but also induced a halt in the cell cycle at the G1 and/or G2/M phases. The effect did not induce death in normal human breast cells, thus ensuring the stable delivery of the effect [450]. The properties of berberine have been attributed to its autophagy-inducing and antioxidant effects. The process of autophagy is regulated by mitochondrial ROS through the activation of AMPK, which in turn upregulates the levels of p-ULK1 that plays a role in autophagosome/lysosome fusion, and beclin1, a well-established regulator of autophagy. The activation and upregulation of these pathways provide evidence that berberine is an effective natural treatment for breast cancer.

#### 5.2.2. Prostate Cancer

Natural product-based nutraceuticals have shown potential in inhibiting growth and promoting apoptosis in prostate cancer cells while preserving normal cells [451]. In particular, several mechanisms are involved in the anti-prostate cancer activity of the molecules, including the inhibition of the androgen receptor axis and targeting of cancer stem cells [235,236].

Several natural molecules, such as quercetin, fisetin, luteolin, apigenin, curcumin, resveratrol, genistein, silibinin, kaempferol, EGCG, tocotrienols, sulforaphane, ginsenosides, ursolic acid, berberine, honokiol, xanthohumol, oridonin, and tannic acid, have shown anti-prostate cancer potential in both in vitro and preclinical experiments [452,453,454].

Curcumin has shown promise in reducing the expression of androgen receptor (AR) and related cofactors including AP-1 [455]. It could control diminishing testosterone production in prostate cancer cells [456]. This is associated with the downregulation of steroidogenic acute regulatory proteins, such as cytochrome P450 11A1 (CYP11A1) and 3-beta-hydroxysteroid dehydrogenase 2, and increased expression of Aldo-Keto Reductase Family 1 Member C2, which eliminates 5α-dihydrotestosterone and inactivates AR [457]. Curcumin inhibits cancer-associated fibroblasts involved in tumor progression and affects microRNAs such as miR-21, leading to the inhibition of cancer growth [458]. The increased expression of miR-21 was found to be significantly and positively correlated with the staging of prostate cancer [459]. In addition, the absence of miR-21 resulted in a substantial decrease in cell growth and hindered the development of prostate tumors in tumor suppressor gene-deleted *Pten/Trp53* mutant mice [460].

Berberine inhibits prostate cancer cell proliferation and reduces the expression of AR, prostate-specific antigen (PSA), COX-2, and B-cell leukemia/lymphoma 2 (Bcl-2) [461]. It decreases the expression of cyclin D1, cyclin-dependent kinase (CDK) 4, and CDK2, and increases the expression of p21 and p27, causing the cell cycle to arrest in the G1/G0 phase. It could also induce apoptosis in various prostate cancer cell lines by targeting the AR signaling pathway. Specifically, it increases the Bcl2-associated X and apoptosis regulator (Bax)/Bcl-2 ratio, promotes the release of cytochrome c, and induces apoptosis through the activation of caspase-3, -8, and -9. Berberine inhibited the activation of EGFR to arrest the cell cycle and hinder cell growth [462]. Moreover, it significantly attenuated prostate cancer cell invasion and migration by downregulating several EMT-related genes [463].

Resveratrol inhibits prostate cancer cell proliferation through various mechanisms including the modulation of nuclear COX-2 accumulation and mitogen-activated protein kinase signaling [464,465], and suppresses metastasis [466]. For example, resveratrol increases the expression of cell cycle inhibitory proteins such as p21 and p27, and decreases the expression of proteins such as cyclin D1, which causes the cell cycle to arrest. Moreover, resveratrol induces apoptosis in prostate cancer cells by regulating the expression of MMP-2 and MMP-9 [467].

Quercetin inhibits the function of AR [468]; reduces the secretion of PSA, HK2, and androgen-regulated tumor biomarkers [469]; and regulates apoptosis in prostate cancer [470]. It inhibits the VEGF-associated phosphorylation of VEGFR2, AKT, and mTOR in prostate cancer and also regulates apoptosis through other mechanisms. It also promotes the expression and phosphorylation of c-Jun in prostate cell lines, inhibiting the activity of PSA and AR promoters [468].

Genistein, a phytoestrogen from soybeans, suppresses AR signaling through ER-β and estrogen-related pathways. In detail, genistein regulates the inhibition of AKT/FOXO3a/GSK-3β and histone deacetylase 6 -heat shock proteins (HSP) 90 function, which are essential for the stabilization of AR. It affects cell proliferation by downregulating the expression of the prostate androgen-regulated transcription factor-1 gene, which is induced by dihydrotestosterone [471].

Natural compounds regulate circadian rhythms in prostate cancer through various mechanisms. Firstly, the disruption of circadian rhythms is linked to tumorigenesis in prostate cancer, emphasizing the importance of integrating circadian rhythm research into prostate cancer studies [229]. Secondly, melatonin, a major circadian regulator, when disrupted in cancer patients, impairs its antioxidant and regulatory functions, making cells more susceptible to mutations and cancer initiation [472]. Additionally, phytochemicals from natural sources have shown anticancer activities by affecting crucial cellular signaling pathways, inducing cell cycle arrest, promoting apoptosis, inhibiting cancer cell growth, and suppressing angiogenesis in prostate cancer cells [473]. These natural compounds target specific pathways like the androgen receptor signaling and prostate cancer stem cells, offering promising therapeutic potentials for prostate cancer management [233].

#### 5.2.3. Lung Cancer

Natural compounds, including phytochemicals, have shown anticancer properties for lung cancer by inducing apoptosis, inhibiting angiogenesis, reversing multidrug resistance, and targeting ROS signaling. These compounds offer potential avenues for the treatment of lung cancer through a multitude of mechanisms, underscoring the significance of investigating natural products in cancer treatment.

Specifically, chaetocin has demonstrated anticancer potential by inducing cell growth inhibition, G2/M phase arrest, and apoptosis in lung cancer cells, making it a potential therapeutic agent [474]. Alkaloids, when incorporated into nano-formulations, can effectively target lung cancer cells by modulating signaling pathways involved in cell multiplication and metastasis [475]. Vinca alkaloids are effective due to their mechanism of microtubule depolymerization and mitotic spindle destabilization, leading to cell cycle arrest and cell death. Camptothecin, found in *Camptotheca acuminate*, forms a complex with topoisomerase, inhibiting its function and causing DNA damage and cell death. Sanguinarine from *Sanguinaria canadensis* suppresses cell growth and induces apoptosis by downregulating the Janus kinase/signal transducer and activator of transcription (JAK-STAT) pathway which mediates downstream events including hematopoiesis, immune fitness, tissue repair, inflammation, apoptosis, and adipogenesis [476], and generates ROS to induce cell death. Liriodenine, sourced from *Cananga odorata*, blocks the G2 to M phase transition of the cell cycle and activates caspases, leading to apoptosis in lung cancer cells. Tambjamine, derived from *Tambja eliora*, disrupts cellular homeostasis, causing mitochondrial dysfunction and lysosomal deacidification, resulting in necrotic cell death.

Natural antioxidants targeting the Nrf2/HO-1 axis have shown efficacy in lung cancer treatment by regulating antioxidant responses [477]. Compounds like theaflavins, quercetin, and curcumin exhibit anti-inflammatory and anticancer properties by suppressing various signaling pathways involved in cancer progression [477]. Resveratrol promotes cell death by increasing pro-apoptotic proteins (Bax and Bak) and decreasing anti-apoptotic proteins (Bcl-2). Moreover, they disrupt cell proliferation by affecting crucial signaling pathways (MAPK, mTOR, PI3K/AKT, and WNT/β-catenin) that control the advancement of cancer. It also functions as a powerful antioxidant, mitigating oxidative stress and protecting cells against DNA damage. Nevertheless, the utilization of resveratrol as the sole approach in cancer therapy is accompanied by limitations, including limited bioavailability, inadequate targeting, and vulnerability to degradation. However, nanoparticle-based delivery systems offer potential solutions to these limitations and have the potential to improve treatment outcomes for lung cancer.

In addition to these, other natural ingredients such as compounds derived from rosemary have been shown to inhibit NSCLC cell bioenergetics and synergize with standard therapeutic drugs [475]. Natural flavonoids including quercetin, EGCG, apigenin, and kaempferol, a group of phytochemicals extracted from plants, have also demonstrated anticancer properties and have the potential to be used as pharmaceutical leads for the prevention and treatment of lung cancer [478].

Natural compounds such as marine-derived agents and green tea polyphenol EGCG have shown potential in targeting circadian rhythms for treating lung cancer. Marine-derived compounds, which are sourced from marine sponges, have been identified as promising anticancer agents due to their cytotoxic effects on cancer cells and targeted effects on specific proteins [479,480]. EGCG, known for its anticancer properties, has been found to repress CLOCK expression in lung cancer stem-like cells, thereby inhibiting their self-renewal ability [480]. Melatonin targets circadian rhythms to prevent and treat lung cancer effectively [481,482]. It is found that melatonin downregulated EMT by inhibiting Twist/Twist-related protein 1 expression, which shows promise in the treatment of lung cancer metastasis [483].

#### 5.2.4. Colorectal Cancer

Research on natural products has shown their potential in targeting colon cancer. These products influence various factors including VEGF, IGF, IL-1, IL-6, COX-2, and chemokines [484,485,486], and can induce the expression of pro-apoptotic proteins while inhibiting anti-apoptotic proteins [487].

Curcumin inhibits TNF-α and NF-κB proteins in malignant cells by targeting the active site of COX-2. It suppresses cell proliferation by enhancing biotransformation enzyme activity and cell cycle protein function [488]. It also reduces HCT-8 cell proliferation by suppressing the PI3K/AKT/mTOR pathway [489]. Additionally, curcumin has been shown to regulate miR-21 expression in CRC, inhibiting invasion and metastasis, highlighting its role in cancer management [458].

Berberine effectively inhibits the proliferation of colon cancer cells, specifically HCT116 and HT29 cells, by downregulating cyclin D1 expression and upregulating the expression of p27 and p21 [490,491]. It also regulates β-catenin in colon cancer cells, demonstrating a dose- and time-dependent inhibition of cell proliferation [492,493].

Resveratrol emerges as a promising therapeutic agent against CRC, exhibiting multifaceted mechanisms to combat tumor progression [494]. It effectively suppresses cancer cell proliferation through the modulation of key molecular targets such as topoisomerase 1 and tyrosyl-DNA phosphodiesterase 1, while inducing apoptosis via ROS-mediated mitochondrial pathways [495]. Resveratrol inhibits invasion and metastasis by reversing EMT and regulating inflammatory responses [496]. Notably, it also enhances the sensitivity of CRCs to other anticancer drugs like cetuximab, thus offering a promising adjunctive therapy [497].

EGCG has been used to effectively inhibit the tumor growth of CRCs. It inhibits the expression of cyclin D1, CDK4, and CDK2, thereby regulating the cell cycle of CRCs and causing the cell cycle to arrest in the G1/G0 phase. It also regulated the hedgehog/phosphoinositide 3-kinase pathways to inhibit proliferation and trigger apoptosis in colon cancer [498].

Luteolin, found in various culinary plants and vegetables, has been demonstrated to possess anticancer activity, specifically against CRCs. It activates the Fas/FasL pathway to induce apoptosis via caspase-8 [499,500]. It also inhibits the development of cancer cells during the G1/S and G2/M phases of the cell cycle. Additionally, it impacts the MAPK and PI3K/AKT pathways, which are involved in regulating cell survive and proliferation [501].

A multitude of other natural products such as protopine [502], vernodalin [503], pyrogallol [504], and ziyuglycoside-II [505], are currently under investigation for their potential to treat and prevent colorectal cancer. These products have been shown to inhibit cancer cell proliferation and metastasis, and induce apoptosis based on various mechanisms.

Melatonin, wogonin, celastrol, and andrographis are natural compounds that have shown promise in targeting circadian rhythms for treating CRC [506]. Melatonin, a hormone regulating circadian rhythm, has demonstrated anticancer effects in combination with irinotecan [506] and *Andrographis paniculate* andrographolide synergistically inhibits CRC cell viability and induces apoptosis [507].

#### 5.2.5. Liver Cancer

Berberine has gained attention for its anticancer properties and as a possible mechanism underlying liver cancer. It inhibits the proliferation of Hep3B and BEL-7404 cells by inhibiting glutamine uptake through solute carrier family 1 member 5 inhibition. It also exhibits a concentration-dependent effect on cell cycle regulation, inducing G1 phase arrest at high concentrations and S phase arrest at low concentrations in HepG2 cells [508]. Berberine’s anticancer activity is further evidenced by its ability to upregulate intracellular ROS levels, downregulate mitochondrial membrane potential, and induce cancer cell apoptosis [509]. Notably, berberine demonstrates synergistic effects when combined with other anti-tumor drugs like sorafenib, leading to the enhanced inhibition of liver cancer cell proliferation and apoptosis induction [510]. Moreover, berberine shows promise in liver cancer prevention by intervening in precursor conditions such as alcoholic fatty liver disease and non-alcoholic steatohepatitis. It achieves this by modulating gut microbiota, exemplified by its ability to increase intestinal farnesoid X receptor and fibroblast growth factor 15 through gut microbiota modulation [511].

Resveratrol has several potential biological effects, including antioxidant, anti-inflammatory, cardioprotective, and anticancer properties, making it effective against liver cancer [512]. It can inhibit cancer cell proliferation and induce cell apoptosis by increasing the expressions of caspase-3, caspase-8, caspase-9, p53, and p21. It can also regulate mRNA and protein expressions such as NF-κB, COX-2, and MMP-2 for apoptosis induction and metastasis inhibition [513]. Furthermore, resveratrol enhances the anticancer effect via inducing G2/M phase arrest and increasing Bx/Bcl-2 ratio [514]. Its capacity to improve the anti-proliferative action of etoposide in CRC HCT-116 and HepG2 liver cancer cells was investigated [515]. Both liver cancer cells express functional p53. Resveratrol turned out to reduce proliferation in both cell lines. While no significant effect was seen in HepG2 cells, resveratrol and etoposide showed better anti-proliferative effects in HCT-116 cells when combined. Furthermore, preincubation with resveratrol raised the level of etoposide-induced p53 in both cell lines and found that levels of p53 were higher following resveratrol and etoposide treatment. These results imply that resveratrol could help to enhance the effects on p53.

Quercetin regulates the signal pathways that are related to the apoptotic process [516]. It activates caspase proteases that are related to cell apoptosis. Caspases are first produced as monomeric, inactive procaspases, which must dimerize and be cleaved frequently to become active. Quercetin could induce liver apoptosis by modulating PI3K/AKT/mTOR and Bcl2/Bax pathways [517]. Along with caspase-dependent cell death, quercetin also affects the caspase-independent Bcl-2 pathway. Specifically, Bcl-2 proteins regulate their interactions through the Bcl homology domain, and quercetin is most active against this Bcl-2 family substrate. In HepG2, quercetin helps regulate Bcl-2 and also increases the proportion of cells in the G0/G1 phase, and inhibits cancer cell survival through the regulation of surviving and Bcl-2 [518].

Matrine is a quinolizidine alkaloid isolated from the roots of *Sophora flavescens* (Kushen), *Sophora tonkinensis*, and *Sophora alopecuroides* (Kudouzi) [519]. Matrine exhibits low toxicity and possesses good water solubility and a diverse range of biological activities, including anticancer, anti-inflammatory, antiviral, and gastroprotective effects. Matrine derivatives, such as benzimidazole matrine derivatives, have shown potent inhibitory effects on DNA topoisomerase I and poly (ADP-ribose) polymerase-1 simultaneously, effectively suppressing cancer cell proliferation [520]. HSPs are molecular chaperones that play crucial roles in tumor development and are increasingly considered as potential therapeutic targets [521]. Rhein, also known as 4, 5-dihydroxyanthraquinone-2-carboxylic acid, is a lipophilic anthraquinone compound that is commonly found in medicinal herbs. These herbs include *Rheum palmatum* L., *Cassia tora* L., *Polygonum multiflorum* Thunb., and *Aloe barbadensis* Miller [522]. Rhein interacts with specific heat shock proteins (namely HSP72, HSC70, and GRP78) in liver cancer cells. This interaction suppresses the function of these proteins and reduces cell viability, suggesting that rhein could potentially serve as a therapeutic agent for liver cancer. Furthermore, when r is co-administered with artemisinin derivatives, there is a significant slowdown in tumor progression in animal models. This indicates that this combination could potentially be an effective therapeutic strategy for liver cancer [521]. Chrysin inhibits the progression of HCC by suppressing the expression of programmed death ligand 1, which is involved in immune evasion by cancer cells, thereby promoting anti-tumor immune responses [523]. Another compound, diosmetin, inhibits the growth and spread of HCC cells by regulating the cell cycle and lipid metabolism pathways, which are crucial for cancer cell proliferation and survival [523]. Autophagic cell death is a process where cancer cells digest their own components, leading to cell death. This process can be triggered by compounds such as berberine and piperlongumine, offering a potential therapeutic approach for liver cancer. Furthermore, the combination of different compounds, such as curcumin and catechin, or biochanin A and SB590885, has been found to have synergistic effects. These combinations enhance the inhibition of tumor progression in hepatocellular carcinoma [523].

Sudachitin, a polymethoxylated flavone found in *Citrus sudachi*, has been identified as a natural compound that enhances liver function through altering circadian rhythms by modulating the amplitude and period of BMAL1 promoter-driven reporter rhythms [524]. Additionally, melatonin, an indoleamine with antioxidant and anti-inflammatory properties, has shown promising effects in modulating circadian rhythms to combat liver cancers, including HCC [525,526]. Melatonin plays a crucial role in regulating circadian rhythms, which is essential for treating liver cancer [525]. It normalizes the expression of circadian proteins like BMAL1 and CLOCK, which are often disrupted in liver cancer cells, aiding in the treatment of cancer. Additionally, melatonin modulates genes involved in the circadian machinery, such as SIRT-1, leading to increased apoptosis in liver cancer cells, indicating its therapeutic potential. By restoring circadian rhythms, melatonin can reduce tumor growth and proliferation, as demonstrated in studies where it decreased the expression of proteins promoting cell division and survival in liver cancer cells [525]. In mice, circadian disruption increases the risk of liver cancer by upregulating oncogenes like c-Myc and downregulating tumor suppressors like p53, which leads to cell cycle activation [527]. Loss-of-function mutations in circadian genes such as *Per2* or *Cry1/2* increase the frequency of chemically induced liver cancer, highlighting the genetic basis of circadian regulation in cancer development [527]. JNK1/2 proteins activate the transcription of the *Bmal1* gene, and this activation influences liver lipid metabolism by promoting lipogenesis. The disruption of JNK1/2 in the liver can lead to bile acid production issues and result in cholangiocarcinoma, indicating their crucial role in linking circadian rhythms with liver health and cancer prevention [528]. Natural compounds like resveratrol, curcumin, and quercetin have shown potential in targeting circadian rhythms to treat liver cancer by modulating clock genes and metabolic pathways [353,445,455].

#### 5.2.6. Gastric Cancer

Natural compounds with therapeutic potential for treating gastric cancer include emetine, quercetin, matrine, and ursolic acid [529]. Emetine, derived from *Psychotria ipecacuanha*, exhibits anti-gastric cancer effects by inhibiting cell growth, inducing apoptosis, and blocking migration and invasion [530]. Quercetin, matrine, and ursolic acid are highlighted in network pharmacology studies as the key components of Shenlian capsule, showing efficacy in inhibiting proliferation and promoting apoptosis in gastric cancer cells through pathways like PI3K/AKT and p53 signaling [529]. Additionally, phytochemicals have been shown to target aerobic glycolysis in gastric cancer cells, affecting pathways like PI3K/AKT, c-Myc, and p53, thereby inhibiting glycolysis, cell proliferation, and migration while promoting apoptosis [531]. Natural compounds like curcumin, crocin, and catechin show promise in treating gastric cancer by inhibiting glycolysis, suppressing proliferation, and promoting apoptosis in cancer cells [532].

Silencing PER1 disrupts the circadian rhythm of PER1-HK2, which controls glycolytic activity in trastuzumab-resistant gastric cancer cells [533]. This disruption leads to the reversal of trastuzumab resistance, indicating that the PER1-HK2 axis plays a crucial role in mediating resistance mechanisms in gastric cancer cells. By targeting PER1, the circadian oscillation of glycolysis is altered, potentially sensitizing the cancer cells to trastuzumab treatment and overcoming resistance. Melatonin inhibits the proliferation of gastric cancer cells through regulating the miR-16-5p-Smad3 pathway, hat circadian rhythms are involved in treating gastric cancer by regulating the miR-16-5p-Smad3 pathway [534]. The identification of miR-16-5p as a key player in melatonin-induced growth inhibition suggests the potential for personalized medicine approaches that target this specific pathway in patients with gastric cancer. However, the objective of reducing gastrointestinal toxicity through the use of circadian rhythms is a promising one, yet further research is required in order to fully comprehend the intricacies of circadian rhythms and develop effective circadian-based interventions that can mitigate gastrointestinal toxicity [535].

#### 5.2.7. Pancreatic Cancer

Curcumin has been demonstrated to possess anticancer properties due to its ability to regulate various pathways and properties. Curcumin regulates the expression of Beclin1, an important protein that regulates autophagy and cell self-destruction, and inhibits the proliferation of pancreatic cancer cells by inhibiting the hypoxia-inducible factor-1α (HIF-1α)-mediated pathway [536,537]. Tumor cells adapt to hypoxia by utilizing glycolysis as an alternative to oxidative phosphorylation to produce adenosine triphosphate (ATP). HIF-1α is a transcription factor that is upregulated under hypoxia. Therefore, reducing it suggests a reduction in the production of tumor cells. Curcumin has been demonstrated to inhibit the proliferation of pancreatic cancer cells under hypoxic conditions, with an inhibitory effect of up to 63.9% observed in PANC-1 cells and up to 63.5% observed in SW1990 cells, depending on the concentration. Furthermore, an increase in curcumin concentration was observed to result in a reduction in the expression of Beclin1 in both the nucleus and cytoplasm. Furthermore, the inhibition of Beclin1 expression was demonstrated to result in a reduction in the binding between Beclin1 and HIF-1α in hypoxic pancreatic cancer cells. These results demonstrated a reduction in ATP production in pancreatic cancer cells by up to 400 pmol/min. The results demonstrated that in hypoxic pancreatic cancer cells, curcumin downregulates Beclin1 expression and inhibits HIF-1α-mediated glycolysis, thereby inhibiting pancreatic cancer cell proliferation.

Ammygdalin is a component found in a variety of fruits and plants, including almonds, peaches, plums, apricots, and apples [538]. The compound exhibits a multitude of efficacious functions against the processes of aging and cancer. These include antioxidant, antibacterial, anti-inflammatory, immunomodulating, and anti-tumor activities [539]. The bitter apricot (*Prunus armeniaca* L.) is a plant belonging to the Rosaceae family and is cultivated in temperate regions around the world, including Central Asia and western China. Bitter apricot seeds exhibit anti-inflammatory, antioxidant, anti-asthmatic, and antibacterial properties. Furthermore, bitter apricot extract has demonstrated anti-tumor activity The bitter apricot extract, prepared using methanol, exhibited an amygdalin content of 314 ± 5.168 mg/g, which was identified as the primary active ingredient. This amygdalin demonstrated cytotoxic effects. These effects have been demonstrated to induce apoptosis in pancreatic cancer cells and to exhibit an anti-tumor effect [540]. When normal cells and pancreatic cancer cells were treated with the extract, the pancreatic cancer cells exhibited apoptotic effects, including fragmented nuclei and apoptotic bodies. However, in normal cells, the morphological apoptotic changes were less evident, and no toxicity was confirmed even after treatment for 72 h. This result demonstrated that the bitter apricot extract was capable of effectively eliminating cancer cells while sparing normal cells. The upregulation of caspase-3 indicates the death of pancreatic cancer cells, and following treatment with the extract, the cells exhibited values comparable to those of the anticancer drug doxorubicin. These findings substantiate the assertion that bitter apricot extract exerts its anticancer effects through a mitochondria-dependent pathway.

## 6. Advantages and Limitations of Natural Compound Use and Future Prospects

Natural therapies, which offer a holistic approach to cancer treatment, address the disease’s underlying causes and bolster the body’s natural healing mechanisms [541]. These therapies serve as an alternative to chemotherapy, which is often hindered by low cure rates caused by multidrug resistance and side effects [542]. Natural compounds, in particular, have shown significant efficacy in both laboratory and animal studies [543].

Compounds found in everyday foods like fruits, vegetables, and spices are especially appealing for their chemopreventive properties and treatment potential. Their widespread consumption, easy availability, high safety margin, and relatively low cost make them an attractive option [544,545]. Certain compounds, including melatonin, curcumin, resveratrol, quercetin, berberine, and EGCG, are known for their antioxidant, anti-inflammatory, autophagy, anti-aging, and anticancer effects, as well as their role in regulating circadian rhythms.

However, the use of natural compounds is not without its challenges. They are not suitable for high-throughput screening due to the difficulties in separating or synthesizing these compounds in large quantities [546]. Despite their high physiological activity, their low solubility leads to poor bioavailability and quick systemic elimination [547]. Many natural compounds are also unstable under physiological conditions or during storage, which can reduce their therapeutic effectiveness [547]. The therapeutic effects of natural compounds can vary greatly depending on the method of preparation and dosage. This variability can make it difficult to standardize treatment protocols [543]. Furthermore, the mechanisms of action of many natural compounds are not fully understood, which can complicate the development of effective treatment strategies, and finally, there are limited scientific and clinical data on their efficacy and safety [543,548]. To improve pharmacokinetic properties, innovative approaches, such as chemical modification [549], the use of drug delivery systems, and nanotechnology, are being developed [141,550]. Rigorous and high-quality clinical trials can help determine the optimal preparation methods and dosages for natural compounds [543]. This can help standardize treatment protocols and ensure consistent therapeutic effects. In addition, high-throughput screening techniques, genomics, proteomics, and metabolomics can be used to enhance our understanding of the mechanisms of action of natural compounds [551]. Advances in biotechnology can facilitate the large-scale isolation of natural compounds [543]. For example, techniques such as microbial fermentation and plant tissue culture can be used to produce natural compounds in large quantities [552]. Collaborations between academic institutions, government agencies, and pharmaceutical companies can greatly promote the development of pharmaceuticals from natural compounds. Implementing policies that encourage the exploration of natural compounds for drug discovery can also be highly beneficial.

The treatment scope with natural products has been broadened using complex combinations, such as Protandim^®^, a supplement containing five natural Nrf2 activators shown to reduce oxidative stress and increase antioxidant enzyme expression [553]. In recent times, combining natural products with chemotherapy has been found to lessen side effects, counteract drug resistance, and enhance treatment results. This approach has proven to be more effective than using natural products alone [543,554].

Chronotherapy, or the timed delivery of drugs in sync with the body’s circadian cycle, aims to enhance treatment effectiveness and reduce toxicity [555]. For instance, in the case of diseases such as asthma, ulcers, and RA, which require treatment at specific times of the day, the use of drug delivery systems with pulsatile drug release, including time-controlled systems and stimulus-driven systems based on various factors such as pH, temperature, and external stimuli, can help ensure correct dosing for patients with chronic diseases, thereby increasing treatment effectiveness [556]. While the application of circadian rhythms in the treatment of diseases holds great promise, it also presents several challenges. Each individual has a unique circadian rhythm influenced by genetics and lifestyle, so it is important to consider the patient’s circadian rhythms when scheduling treatments. This ties into the concept of personalized approaches. Further research is needed to fully understand the implications of circadian rhythms on health and disease, as well as to overcome the obstacles associated with their implementation in clinical practice. However, the potential benefits of this approach make it a promising area for future healthcare innovations [557].

Lifestyle improvements that align with circadian rhythms can effectively lower cancer risk and improve the health of cancer survivors [33,34]. These improvements include adequate sleep, time-restricted eating, daylight exposure, and optimal exercise timing. By leveraging circadian rhythm principles, we can potentially revolutionize cancer prevention, prognosis, and survival [558]. However, more research is needed to fully harness the potential of these approaches [555].

## 7. Conclusions

Overall, the use of natural compounds and herbal medicines shows great potential in both preventing age-related diseases and slowing down the progression of cancer. Their modes of operation, encompassing antioxidant characteristics, anti-inflammatory impacts, cell proliferation inhibition, autophagy activation, apoptosis promotion, and the suppression of metastasis and angiogenesis, play a vital role in disease prevention. The significance of circadian rhythms in disease risk is also notable, as disrupted rhythms impact the gene expression related to cancer and inflammatory diseases. Compounds such as melatonin and certain phytochemicals including berberine, curcumin, EGCG, quercetin, and resveratrol have the ability to regulate these rhythms, thereby managing inflammation, immune responses, and redox homeostasis. These factors play a crucial role in preventing age-related diseases and impeding the advancement of cancer. Although natural compounds face challenges in their effectiveness for cancer treatment, such as low bioavailability, possible interactions with other treatments, the lack of standardized formulations, and limited scientific and clinical data on efficacy and safety, these obstacles can be overcome. The future of natural compounds in disease prevention and cancer treatment appears promising due to the increasing availability of clinical data that support their efficacy and safety in treating chronic diseases. Furthermore, advancements in nanotechnology are enhancing the bioavailability and durability of these compounds. As we further investigate and comprehend the intricate mechanisms of aging and cancer, which are affected by genetics, lifestyle, and environmental factors, the significance of natural compounds and herbal medicines will inevitably grow in our pursuit of good health and long life.

## Figures and Tables

**Table 4:** Major plant ingredients used for medicinal purposes.

Group	Description
Alkaloids	Nitrogen-bearing molecules found in a variety of plants. Used in drugs like vincristine (from Madagascar periwinkle) and sinomenine (from *Sinomenium acutum*) [325] for cancer treatment; atropine (from deadly nightshade) for emergency medicine, anesthesia, cardiology, and other specialties as well as reversing bradycardia and managing organophosphate poisoning [326]; and morphine (from the poppy plant, *Papaver somniferum*) for pain relief in cancer patients [327].
Bitters	Plants with a bitter taste that stimulate salivary glands and digestive organs. Examples include hop acids from the hop plant (*Humulus lupulus*) exhibiting anticancer activity [328], and amarogentin (*Gentiana lutea radix*, L.), a bitter taste receptor activator regulating a variety of cell signaling including AMP-activated protein kinase (AMPK), STAT3, Akt, ERK, and p53 [329]
Cardiac glycosides	Compounds (digitoxin, digoxin, and convallotoxin) in medicinal plants like Foxglove and Lily of the Valley that support heart strength and stimulate urine production.
Cyanogenic glycosides	Glycosides based on toxic cyanide found in plants like wild cherry and elderberry. Used in small doses as a muscle relaxant and to soothe dry coughs.
Flavonoids	Compounds found in many plants that often act as pigments and have antiviral and anti-inflammatory properties. They generally have a 15-carbon skeleton and are known to strengthen capillaries and prevent leakage into tissues. Examples include lemon and buckwheat.
Minerals	Many plants draw minerals from the soil and convert them into a form easily used by the human body. Example: horsetail, which is high in silica and used for arthritis because it supports the repair of connective tissue.
Phenols	Plant compounds thought to protect against infection and herbivory. Often anti-inflammatory and antiseptic. Phenols vary in structure and range from salicylic acid to complex sugar-containing phenolic acids. Examples include wintergreen, willow, and mint family.
Polysaccharides	Sugar molecules found in all plants that can form jelly-like masses used to treat dry or irritated tissues.
Proanthocyanins	Pigments related to tannins and flavonoids that protect circulation. Found in red grapes, blackberries, and hawthorn berries.
Saponins	Active compounds that produce lather in water. Found in plants like agave and wild yam. Steroidal saponins are very similar to the chemical structures of many of the human body’s hormones including estrogen and cortisol. Some of them are used to produce synthetic hormones.
Tannins	Compounds produced by most plants that deter herbivory and are useful in curing leather. Found in plants like oak bark and black catechu.
Vitamins	Many plants contain high levels of useful vitamins. Examples include watercress, rose hips, and sea buckthorn, which have high levels of vitamins B, C, and E.
Terpenes/terpenoids	Terpenes and terpenoids are the main bioactive compounds of essential oils, volatile and concentrated liquids extracted from different parts of plants. Example: tea tree oil, a strong antiseptic. Carvacrol, carvone, eugenol, geraniol, and thymol for antimicrobial activity [330]. Paclitaxel (taxol from *Taxus brevifolia* tree) for cancer treatment [331]
Fatty acids	Omega-3 fatty acids play an important role in regulating brain function and lowering cardiovascular disease risk; alpha-linolenic acid from nuts and seeds, flaxseed oil, soybean oil, and canola oil [332]; eicosapentaenoic and docosahexaenoic acid from microalgae [333]

The table was adapted from the webpage (source: U.S. Department of Agriculture; U.S. Forest Service: Active plant ingredients used for medicinal purposes) [334].

## Data Availability

The data presented in this study are available in this article [http://doi.org/10.3390/ijms25147530], reference number [14,38,56,57,204,205,334]. Table 1, Table 2 and Table 3 were derived from the following resources available in the public domain: Estimated deaths by cause, sex, and WHO member state, 2019, and https://www.who.int/data/gho/data/themes/mortality-and-global-health-estimates/ghe-leading-causes-of-death (accessed on 19 April 2024); Age-Standardized Rate (World) per 100,000, Incidence and Mortality, Both sexes, in 2022 World, In Cancer Today and https://gco.iarc.fr/today/en/dataviz/tables?types=0_1&mode=cancer&group_populations=1&sort_by=value1&multiple_populations=1&populations=900&sexes=0 (accessed on 7 June 2024).
